# Social processes and engagement along the HIV care continuum: a mixed methods exploratory study with diverse African American/Black and Latine emerging adults living with HIV

**DOI:** 10.1186/s12939-025-02662-5

**Published:** 2025-10-28

**Authors:** Leo Wilton, Marya Gwadz, Charles M. Cleland, Michelle R. Munson, Stephanie Campos, Khadija Israel, Maria Medvedchikova, Nisha Beharie, Corey Rosmarin-DeStefano, Dawa Sherpa, Samantha Serrano

**Affiliations:** 1 Department of Human Development, State University of New York at Binghamton, 4400 Vestal Parkway East, Binghamton, NY 13902 USA; 2 Faculty of Humanities, University of Johannesburg, PO Box 524, Auckland Park, Johannesburg, 2006 South Africa; 3 Silver School of Social Work, New York University, 1 Washington Square North, New York, NY 10003 USA; 4 Department of Population Health, New York University Grossman School of Medicine, NYU Langone Health, 180 Madison Avenue, 2-53, New York, NY 10016 USA; 5 Independent Consultant, Jersey City, NJ 70304 USA; 6 School of Global Public Health, New York University, 708 Broadway, New York, NY 10003 USA; 7 Department of Public Health, School of Health Science and Practice, New York Medical College, 30 Plaza West, Room 223, Valhalla, NY 10595 USA; 8 North Jersey Community Research Initiative, 393 Central Avenue, Newark, NJ 07103 USA

**Keywords:** HIV care continuum, African American/Black, Latino/Latine, Emerging adults, Social factors, Social networks, Immigrants, Disparities, Substance use, HIV viral suppression

## Abstract

**Background:**

Racial/ethnic and age-related disparities in HIV care continuum engagement are serious in the US. American/Black and Latine (AABL) young/emerging adults living with HIV have the lowest rates of engagement, but aspects of their experiences and some subpopulations are understudied. The present study is grounded in social action theory and uses a sequential explanatory mixed methods design to explore social interaction processes (e.g., social networks, trust), and their relationships to HIV management, in a diverse cohort including those with non-suppressed HIV viral load.

**Methods:**

Participants engaged in baseline assessments (*N* = 271). A subset (*N* = 62) was purposively sampled for maximum variability for qualitative interviews. Quantitative data were analyzed with descriptive statistics and logistic regression. The primary outcomes were engagement in HIV care and viral suppression. Quantitative results were used to develop qualitative research questions, and qualitative data were analyzed with directed content analysis. Joint display methods were used to integrate results.

**Results:**

On average, participants were 25 years old (SD = 2). The majority (59%) were Latine/Hispanic, 41% were African American/Black. Nearly all were male (96%) and sexual minorities (93%). Nearly half (49.1%) were born outside the US/Puerto Rico. Most were well-engaged in care (72%) and virally suppressed (81%). The proportion of network members who used illicit drugs reduced the odds of being well-engaged in HIV care (OR = 0.38; *p* = 0.014) and suppression (OR = 0.32; *p* = 0.009) at the bivariate level. Satisfaction with HIV care (OR = 1.18; *p* = 0.016) and trust in providers (OR = 1.04; *p* = 0.045) increased the odds of viral suppression. In qualitative results we found social networks were vital to well-being, but, like participants, located in strained socioeconomic circumstances. In this context, we organized results into five themes: (1) social losses were disruptive to HIV management; (2) service settings and care providers were important network members; (3) family disapproval of sexual/gender minority status had negative effects; (4) immigrant participants were highly reliant on service settings; and (5) networks influenced participants’ drug use and their drug use also reduced the size and changed the composition of networks.

**Conclusions:**

The present study advances knowledge on social interaction processes among diverse AABL young/emerging adults living with HIV, and highlights points of intervention.

**Supplementary Information:**

The online version contains supplementary material available at 10.1186/s12939-025-02662-5.

## Introduction

The United States (US) public health system intends to end new HIV diagnoses by the year 2030 [1]. To do so, 95% of people living with HIV need to know their status, 95% of diagnosed individuals need to take HIV medication, and 95% of those on HIV medication must maintain use at high levels of adherence to achieve and sustain HIV viral suppression [2]. These are called the 95-95-95 targets [2, 3]. While rates of engagement along this HIV care continuum have increased in the past decade, disparities related to age and race/ethnicity are serious and persistent [4, 5]. Regarding disparities across age groups, younger people living with HIV consistently fall short of these 95-95-95 objectives and evidence lower rates of engagement than their older peers [6]. For example, among those ages 13–24 years diagnosed with HIV across all racial/ethnic groups, 80% have received HIV care, 55% are retained in care, and 65% are virally suppressed based on their most recent test [6]. Rates of engagement are similarly insufficient for the next highest age group tracked by the Centers for Disease Control and Prevention (CDC), those aged 25 to 34 years [6]. Regarding racial/ethnic disparities across all age groups, African American/Black and Latine (AABL) persons are overrepresented among the population of people living with HIV compared to their proportions in the general population, but overall less likely to engage in HIV care and take HIV medication with high levels of adherence compared to their White counterparts [4, 5]. Among younger people living with HIV, compared to their White peers, AABL younger people living with HIV ages 13–24 years are less likely to be virally suppressed (White 72%, African American/Black 63%, Latine 70%), with similar disparities evident in the 25 to 34-year age group [7]. Racial/ethnic disparities in rates of sustained HIV viral suppression are also marked, where AABL young and emerging adults evidence lower rates of sustained HIV viral suppression than their White counterparts (White 51%, African American/Black 36%, Latine 47%) [8]. Research on AABL younger persons is needed to reduce or eliminate these persistent disparities, including with subpopulations that are understudied such as those with non-suppressed HIV viral load, monolingual Spanish-speaking and immigrant, refugee, and asylum-seeking persons, and young people experiencing severe socioeconomic barriers [9, 10]. We refer to the age group as “emerging adults” throughout the rest of this paper for parsimony.

Emerging adulthood is a developmental period of transition and transformation [11–13]. AABL emerging adults living with HIV experience developmental challenges typical of this period, in domains such as finances, housing, and self-directed management of roles and responsibilities. They also face atypical undertakings inherent in ongoing HIV management, along with associated difficulties such as experiences of stigma and discrimination related to race/ethnicity, sexual orientation, gender identity (in some cases), and HIV status [14, 15]. Emerging adulthood is also a time of change in social relationships, both peer and family, primary among them the reorganization of social ties in response to increasing autonomy, the renegotiation of parent-child relationships, and the potential for romantic relationships to lengthen and solidify [11–13]. In the context of this developmental period of transition, social networks play a crucial role in HIV management, as discussed below. Leaders in the field have called for expanding scientific frameworks to include the social network [16–18]. However, few studies of the social networks of AABL emerging adults living with HIV are found in the empirical literature. The present study addresses this gap.

**Fig. 1:**
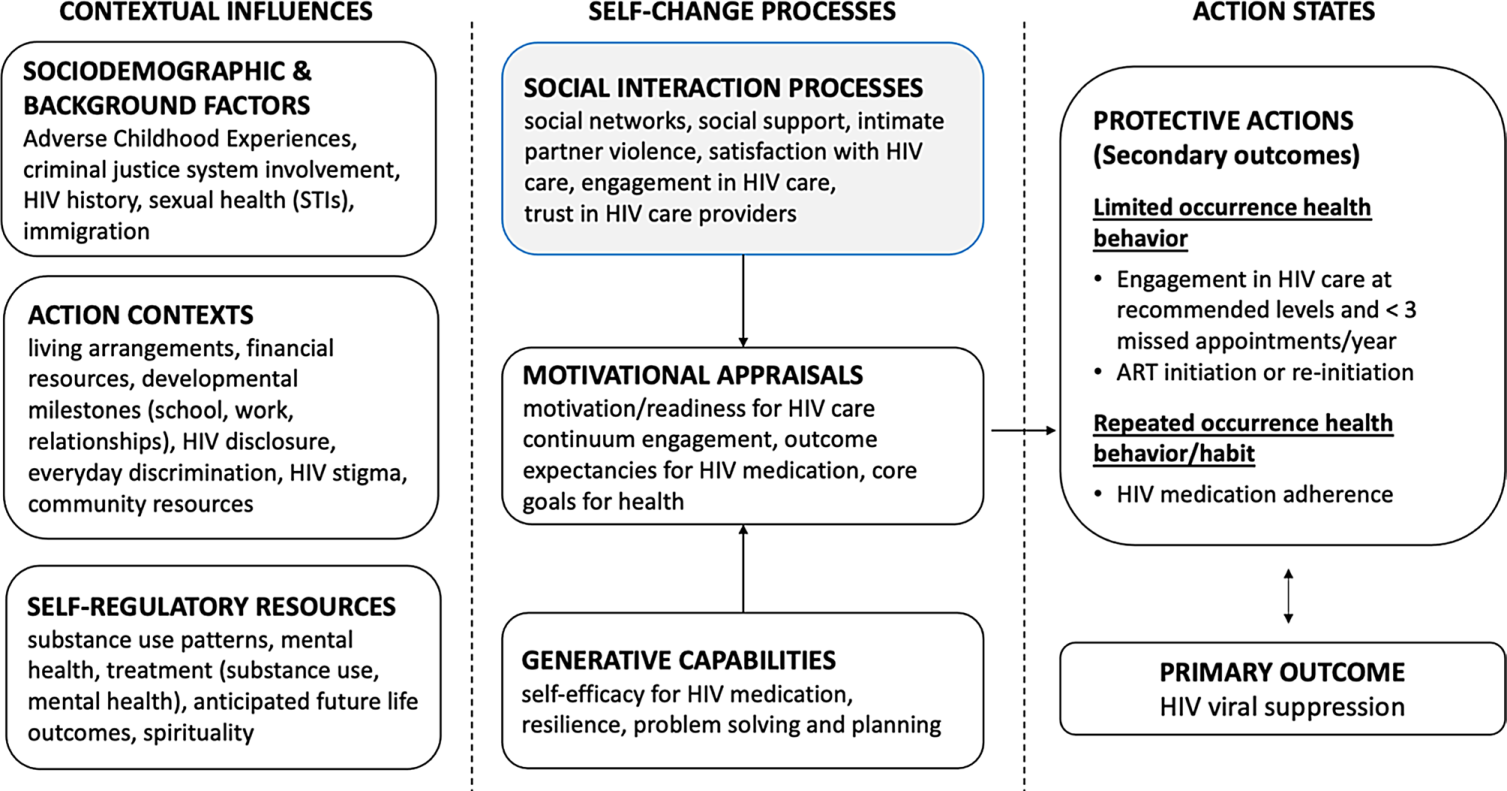
Social action theory model with the domains examined in the present study highlighted

Social action theory, a comprehensive social-cognitive model, guides the present study [19]. Social action theory describes how upstream contextual influences (specifically, background factors, action contexts, and self-regulatory resources) influence self-change processes at the social and individual levels, which then in turn produce action states (protective actions) that lead to health outcomes, such as HIV viral suppression (see Fig. 1). These contextual influences and self-change processes may have direct effects on action states and health protective actions. They may also influence other domains in the model, such as important mediating factors (e.g., motivation). In past research, social action theory has been adapted for use with persons living with HIV and young men who have sex with men [20, 21]. We modified social action theory for diverse AABL emerging adults living with HIV to study HIV management in this population. In adapting the theory, we took into consideration developmental challenges typical of emerging adults and atypical difficulties the population faces, including ongoing HIV management and the psychosocial considerations that influence or arise from HIV, such as stigma, disclosure, and medical distrust [11–13].

In two previous papers on this cohort of diverse AABL emerging adults living with HIV, we explored contextual influences (e.g., adverse childhood experiences), and self-regulatory resources (e.g., substance use management, mental health) [9, 22]. In the present study, we explore one aspect of self-change processes, namely, social interaction processes. Social action theory recognizes that an individual’s social contexts and relationships influence motivational appraisals (such as core goals for health) and generative capabilities (such as self-efficacy), as well as health-related actions and behavior change efforts [19]. Figure 1 highlights the domains explored in this paper. We briefly review the literature on these social interaction process domains in the section that follows.

AABL emerging adults living with HIV are embedded within social networks that include family, friends, romantic and sexual partners, health care providers, and others. Social network members can be characterized with respect to their relationship to the participant, length and closeness of relationship, sociodemographic characteristics, risk and protective factors (e.g., school, work), and HIV status, referred to as an egocentric perspective [16, 18, 23]. Navigating emerging adulthood and living with HIV requires meaningful and consistent social support that is often available only from close confidants [24], and these social connections and interactions play an important role in HIV care continuum engagement and general well-being [18, 25, 26].

Characteristics of social network members can influence AABL emerging adults living with HIV, and vice versa. Primary among these is substance use among social network members. In the general population, an individual is more likely to use substances when friends and family implicitly or explicitly encourage use, and if they have close peer network members who use substances [27, 28]. Further, intimate partner violence, one aspect of social relationships, can have serious adverse effects on physical and mental health [29, 30]. Intimate partner violence is associated with negative health and behavioral outcomes among persons living with HIV and can disrupt HIV medication adherence [30].

As shown in Fig. 1, social interaction processes also include aspects of the HIV care experience and relationships with providers. Satisfaction with HIV care is associated with HIV medication adherence and appointment attendance, for example, when the setting is experienced as competent and responsive to needs [31, 32]. Relatedly, engagement in HIV care, defined as the extent to which individuals are actively involved and invested in their own healthcare and experience a collaborative partnership with their providers, is associated with positive HIV outcomes [33, 34]. Trust in health care providers is another important aspect of engagement along the HIV care continuum [35, 36]. However, AABL persons in the general population report lower levels of trust in their healthcare providers compared to White persons, linked to factors such as perceived discrimination, historical injustices, and disparities in access to care [37]. These findings may extend to persons living with HIV, although some studies have not found racial/ethnic differences in associations between trust and HIV care continuum engagement [38–40].

## Methods

The present study uses mixed methods and a sequential explanatory mixed methods design [41] to explore social interaction process domains that may influence HIV management for this population, which is diverse and includes subgroups that are understudied in research. It draws on quantitative baseline data and qualitative data from a larger longitudinal study focused on diverse AABL emerging adults living with HIV aged 19–28 years, including those with and without HIV viral suppression (*N* = 271 individuals, 19% with non-suppressed HIV viral load). The larger study was conducted in New York City and Newark, New Jersey (NJ), and carried out by an academic institution in New York City in partnership with the North Jersey Community Research Initiative, a large multi-service community-based organization in Newark, NJ. Participants were enrolled between December 2021 and October 2023. A subset of participants (*N* = 62) was selected for semi-structured interviews. The qualitative study component took a phenomenological approach to uncover participants’ views on the causes, meanings, and effects of social processes on HIV management [42]. Quantitative interviews took place in person and qualitative interviews were conducted either in person or on the phone. In person study activities took place mainly at a field site in lower Manhattan but also in Newark, NJ. Activities were carried out in English and Spanish. We compensated participants for study activities. The study was approved by the Institutional Review Board at New York University, and participants gave informed consent. Methods are detailed elsewhere [9, 10] and reviewed briefly below.

### Design

The sequential explanatory mixed methods design proceeds in phases: first, quantitative data are analyzed. Then, a set of research questions is developed based on the quantitative findings, to add richness, meaning, and context to the findings, and qualitative data are analyzed [41]. Last, quantitative and qualitative data are integrated [41]. For the present study, we used quantitative data to describe social interaction processes among the sample as a whole and the subgroups of those HIV virally suppressed and those who are not, followed by an examination of the relationships between these factors and two outcomes: being well-engaged in HIV care and HIV viral suppression. Second, we developed qualitative research questions in response to these findings, focused on social networks. Last, quantitative and qualitative results were integrated using the joint display and themes-by-statistics methods [43]. The analysis plans are described in more detail below.

### Eligibility for the parent study

Eligibility criteria for the parent study were: age 16–28 years; AABL race/ethnicity; residence in the New York City or Newark, NJ metropolitan areas; HIV diagnosis (confirmed with medical documentation); diagnosed with HIV *≥* 3 months ago; HIV was transmitted behaviorally, not perinatally; and able to conduct activities in English or Spanish. HIV viral load testing from a commercial laboratory or a recent laboratory report from their primary care setting was obtained to assess viral suppression status at enrollment, but this was not an eligibility criterion.

### Recruitment for the parent study

Participants were recruited for the parent study with a hybrid strategy that included a peer-to-peer approach, social media, dating apps, ads placed in public transportation venues, and recruitment in community-based organizations in New York City and Newark, NJ [10]. Emerging adults are challenging to engage in research, due to competing priorities, lack of experience in making independent decisions about research, and/or not prioritizing the need for research participation [44, 45]. Similar to the general population of emerging adults, AABL emerging adults living with HIV are challenging to engage in research, particularly those poorly engaged along the HIV care continuum, since most research with this population is conducted in medical settings [9, 10, 22, 46–48]. The present study took the approach of recruitment in the community rather than medical settings to enroll a diverse cohort, including those with barriers to medical locations and research [49, 50]. In particular, we targeted those with non-suppressed HIV viral load, because of the importance of HIV viral suppression for individual and public health, as noted above. We also sought to locate and engage other understudied subpopulations, such as monolingual Spanish-speaking and immigrant, refugee, and asylum-seeking persons, and young people experiencing severe socioeconomic barriers [9, 10]. This community-recruitment approach complements studies conducted in medical settings to advance knowledge of this population.

### Types of qualitative interviews

Qualitative interviews took one of two approaches: in-depth qualitative (*N* = 41) or qualitative life history (*N* = 21). Both types of qualitative interviews took a phenomenological approach, a method to understand the lived experiences of individuals and the meanings they assign to those experiences [42]. At the start of the project, we carried out in-depth qualitative interviews. However, although most interviews tended to be long and detailed, it could be challenging for some participants to articulate relationships between life events and HIV management experiences. In response to this challenge, we added the qualitative life history format to the study. This latter format was more structured than the former, since participants would focus on a discrete period of time in their lives. When a participant was selected for an interview, we alternated between the two types. However, we did not anticipate that the type and quality of data would differ *substantially* between the two interview formats, because were designed to elicit participants’ experiences and the meaning they ascribed to these experiences. Thus, the aim of using two qualitative interview approaches was to generate a comprehensive and detailed data set, focused on complex phenomena that can be challenging for some participants to understand and articulate. The data (interview transcripts) were combined into a single data set.

### Sample size for the qualitative interviews

The sample size (*N* = 62) was determined following guidelines from Malterud and colleagues called “information power” [51], which consider characteristics of the study and sample specificity. Since the domains under study were complex and numerous, as shown in Fig. 1, and the sample was expected to be diverse, we opted for a larger sample size.

### Procedures

#### Screening for eligibility and baseline assessment

We obtained verbal informed consent, and then potential participants were screened for eligibility. Among those found eligible, participants provided signed informed consent. Then, the structured baseline assessment battery (taking 60–90 min) was carried out ($60 compensation).

#### Sampling for the present study

Participants were selected from the larger sample for qualitative interviews. For both types of qualitative interviews, participants from the larger cohort were selected using purposive sampling for maximum variability on key indices, namely race/ethnicity, language (English vs. Spanish), and HIV viral load status (suppressed vs. non-suppressed).

#### Qualitative in-depth interview procedures

As noted above, both types of interviews were phenomenological in nature, but used different interview formats. In-depth qualitative interviews were guided by a template organized by key questions, focused on understanding the participant as a whole person, important aspects of the participant’s life (e.g., family acceptance, immigration experiences, gender affirmation), and factors that promoted or impeded their engagement along the HIV care continuum and their causes and meanings (*N* = 41). In the life history interviews, participants were asked to reflect on specific sequential periods of their lives with an aim of understanding these experiences (*N* = 21). (More information on the interview templates is provided below, and the templates are attached as supplemental material.) Thus, a total of 62 qualitative interviews were conducted, approximately a quarter in Spanish (24%, 15/62). The interviews were conducted by phone or in person between six and twelve months post-enrollment, and lasted between 60 and 90 min. Participants were compensated $60 for the qualitative interview. Interviews were conducted by four Master’s and PhD-level qualitative researchers trained in anthropology or public health, three of whom were fully bilingual in Spanish and English. Interviews were audio-recorded and professionally transcribed. Those conducted in Spanish were translated into English by a professional service, and then transcripts were checked for accuracy by members of the research team who were fluent in Spanish.

### Measures

#### Sociodemographic characteristics and background factors

Demographic characteristics included age (in years), years since HIV diagnosis, sex assigned at birth (male, female, intersex, other), gender identity (coded as cisgender vs. transgender, gender expansive, gender non-binary, gender queer or otherwise not cisgender, yes/no), sexual orientation (coded as gay/homosexual vs. other identities, yes/no), race/ethnicity, primary language (English or Spanish), education (coded as less than high school vs. high school graduate or higher), and insufficient income (could not meet needs for necessities such as rent, food, utilities in past six months) [52, 53]. We assessed the presence or absence of dimensions of childhood adversity prior to the age of 18 years using a 14-item version of the Adverse Childhood Experiences inventory called the ACES-R (e.g., emotional abuse, physical abuse, sexual assault, emotional neglect, physical neglect) [54]. ACES items were coded on a yes/no scale and the sum of affirmative responses was calculated, which comprised the ACES-R score ranging from 0 to 14 (Cronbach’s α = 0.89).

### Social interaction processes

#### Social networks

To describe the size and composition of social networks, we used an egocentric approach taken by da Silva and colleagues, modified for this population [16]. This approach focuses on the social connections of a single individual, the participant (the “ego”), and their direct relationships (the “alters”) [16]. Participants were asked to list up to five members of their close and personal social network, defined as “people with whom you discuss things that are important to you.” After populating the network, participants described for each network member the type of relationship (friend, romantic partner, sexual partner, family member, or other); age (in years); sexual orientation (e.g., gay, lesbian, straight, bisexual, other); gender identity (e.g., man/male [cisgender], woman/female [cisgender], gender non-binary, genderqueer, gender expansive, transgender or similar term [non-cis-gender], recoded as cisgender male, yes/no); race; ethnicity; HIV serostatus; if diagnosed with HIV, whether takes HIV medications most of the time; criminal justice system involvement; use of illicit drugs; currently enrolled in school or a training program; currently working in the formal economy; how long the participant has known the person (in years); and how often they see each other (daily, weekly, monthly, occasionally, recoded as seen network member daily or weekly, yes/no). Data were coded into the following variables: The mean and standard deviation (SD) were calculated for the size of networks (range 0–5), years known to each other, and the age of network members. For the following characteristics, the proportion of members in each category was calculated (mean, SD): cisgender male, sexual and/or gender minority, African American/Black race, diagnosed with HIV, takes HIV medication most of the time, uses illicit drugs, currently in school or college, and currently working in the formal economy [16].

#### Social support

We used a 9-item measure, the MOS Social Support Survey, modified to assess support in the context of living with HIV. The measure assessed functional social support in multiple dimensions: emotional/informational, tangible, affectionate, and positive social interaction (e.g., If you needed it, how often is someone available who understands your problems?, If you needed it, how often is someone available to talk about what it’s like to live with HIV?) [55]. Items were assessed on a 5-point Likert scale (none of the time, a little of the time, some of the time, most of the time, all of the time). An overall support index was calculated on a 0-100 scale where a higher score indicates greater levels of social support (Cronbach’s alpha = 0.91).

#### Intimate partner violence

Intimate partner violence was assessed with four items answered yes or no, focused on the lifetime and the past six months: “Have you been slapped, punched, kicked, beaten up, or otherwise physically or sexually hurt by a partner, boyfriend/girlfriend, spouse, or some other intimate partner?;” and “Have you ever slapped, punched, kicked, beaten up, or otherwise physically or sexually hurt a partner, boyfriend/girlfriend, spouse, or some other intimate partner?” [56].

#### Satisfaction with HIV care

Satisfaction with the location where the participant received most of their primary care services was assessed with two items, each rated on a five-point Likert scale ranging from 0 (poor) to 4 (excellent). The two items were: 1. “Overall, you think that the services there are poor, fair, good, very good, or excellent” and “The information that you have received there has been poor, fair, good, very good, or excellent.” Scores were summed and ranged from 0 to 8 with higher scores indicating greater satisfaction with HIV care.

*Engagement in HIV care;* that is, participants’ expectancy regarding and investment in HIV care, was assessed with an eight-item measure using a five-point Likert scale (strongly disagree to strongly agree) [34]. Focused on the primary setting where participants received HIV primary care, items included: “I am fully invested in making use of the services [setting] is providing me” and “the [setting] is truly helping me take care of some problems in my life.” Items were reverse-coded as needed and summed (range 0–32), and higher scores indicate greater engagement in HIV care (Cronbach’s alpha = 0.82).

*Trust in HIV care provider* was assessed with an 11-item scale [57]. Items included “I trust my health care provider to put my medical needs above all other considerations when treating my medical problems” and “My health care provider is a real expert in taking care of medical problems like mine.” Items were assessed on a five-point Likert scale (strongly disagree to strongly agree). Items were reverse-coded as needed and summed (range 0–44), and higher scores indicate greater trust in the HIV care provider (Cronbach’s alpha = 0.88).

### Primary outcomes (HIV care continuum indices)

We assessed whether participants were well-engaged in HIV care (yes/no) [58, 59]. This variable was comprised of two indices: whether two or more HIV primary care appointments were attended in the past year, an accepted minimum [60], and whether the participant had not missed three or more HIV primary care appointments past year without prior cancellation, as missed visits are independently associated with mortality [61]. HIV viral load was assessed via laboratory report and coded on the log_10_ scale [62]. We present the mean and SD and also coded HIV viral load status as suppressed (*≤* 200 copies/mL) or non-suppressed (>200 copies/mL) [62].

### Qualitative interview template

Semi-structured templates guided the qualitative interviews. The templates were developed by the research team, which included experts on the HIV care continuum, young and emerging adults, AABL persons living with HIV, sexual and gender minorities, and immigration, along with a community advisory board made up of AABL young and emerging adults living with HIV. The in-depth interview template was structured as a series of questions and prompts, starting with more general questions and then moving to more specific questions within a number of domains. The main purposes of the guide were to understand the participant as a whole person, understand factors that promote or impede engagement along the HIV care continuum and their causes and meanings, and explore domains that were not included in the structured assessments but that might be important. The life history template was structured around five developmental periods (0–5 years, 6–12 years, 13–16 years, 17–21 years, and 22 years – present). Within each period, participants were asked about major life events experienced and their contexts, causes, and meanings. In a second step, the interviewers attended to HIV medication stops and starts, and the context of stops and starts, within the periods post-diagnosis. Third, participants were asked to reflect on the issues raised across the life course.

### Quantitative data analysis

We used descriptive statistics to summarize HIV viral suppression and care engagement outcomes, as well as sociodemographic and background characteristics, HIV and other health history, and social interaction processes. We present data for the cohort as a whole, and for the HIV virally suppressed and non-suppressed subgroups. Because of the large number of comparisons, we did not conduct tests of significance for the descriptive variables. They are presented for descriptive purposes.

Sixteen participants (6%) did not list any social network members. An additional 20 participants had missing data on intimate partner violence (*n* = 5), social support (*n* = 4), HIV care satisfaction (*n* = 9), HIV care engagement (*n* = 9), or provider trust (*n* = 9). Missing data were addressed under the assumption they were missing at random (MAR) conditional on observed data by employing multiple imputation. The R mice package [63] was used to impute 10 datasets by predictive mean matching. Results from each imputed dataset were pooled using Rubin’s rules [64].

To estimate associations between sociodemographic and background factors, HIV and other health factors, social interaction processes, and the two outcomes, we used binary logistic regression. For each outcome, we estimated bivariate associations as well as associations adjusted for all other variables in the model. Coefficients estimated by binary logistic regression are log odds ratios, and exponentiating the coefficients leads to odds ratios (ORs), which describe how a one-unit change in the explanatory variable multiplies the outcome variable odds. Associations were reported as ORs with 95% confidence intervals. The R statistical computing program [65] was used for all analyses, including multiple imputation, tests of significance, and confidence intervals. All tests of statistical significance were two-tailed, and *p* < 0.05 was considered significant.

### Developing the qualitative research questions in response to the quantitative findings

After conducting the quantitative analyses, the research team used the findings to generate a set of research questions that could contextualize and advance our understanding of those findings, and that could be reasonably addressed using the qualitative data. For parsimony, we attended to a select set of quantitative findings, attending to domains understudied in the literature. We focused on social networks and developed a question on social networks generally; namely: how do social networks support or impede HIV management, and how do systemic/structural and social-level factors influence the effectiveness of the network to support HIV management and well-being generally? We also developed a second and more specific question about networks in response to the quantitative findings showing that the proportion of network members who used illicit drugs was associated with a lower odds of being well-engaged in HIV care and with HIV viral suppression (Tables 3 and 4); namely, what are participants’ perspectives on drug use in their social networks and its effects on HIV management and well-being generally?

### Qualitative data analyses

Analyses of qualitative data followed a directed content analysis approach that was both theory-driven and inductive [66]. Analyses were carried out in Dedoose, a cross-platform application for analyzing qualitative and mixed methods research. We started with an initial list of “start codes” and their operational definitions generated by the primary qualitative analyst (SC). This start code list was informed by social action theory and the goals of the larger study. Using this code list, two analysts coded interview transcripts (SC and NB). During the coding process, codes were refined, clarified, and/or broadened. Discrepancies in codes and coding between the data analysts were resolved by consensus. Interview transcripts were recoded using the final coding frame. We then formed an interpretive community to organize codes into themes and sub-themes in an iterative process. The interpretive community was led by the primary analyst (SC) and included members of the research team (MG, NB, MRM, MM, DS, and SS). Methodological rigor of the analysis was monitored using an audit trail of process and analytic memos [67]. The primary analysts and the interpretive community attended to the potential effects of the team’s positionality related to power and privilege, sex, gender, race/ethnicity, citizenship status, health, and socioeconomic status throughout the data collection process through reflection and training that focused on how these factors might affect interviewing and data analytic processes [68, 69].

### Data integration procedures

We used two methods to integrate the quantitative and qualitative results. First, the joint display method was employed following procedures described by Fetters and colleagues [43]. A joint display is a visual tool that consists of a side-by-side visual presentation of results. The process of creating the joint display is intended to bring about new insights beyond the information gained from the separate quantitative and qualitative results. Thus, joint displays are both a method and a cognitive framework for data integration and facilitating the production of new inferences [43]. Data integration was carried out by the research team and led by one of the study’s principal investigators (MG). First, beginning with the major quantitative findings, the research team assessed areas of convergence and divergence between the quantitative results and the primary themes in the qualitative data analysis. To do so, an informational matrix was used to compare results at a granular level (major finding-by-finding). The results from this data integration effort were summarized and presented in a joint display table. Second, we carried out a themes-by-statistics analysis. Analysts created a themes-by-statistics display focused on social networks, drug use, and HIV treatment engagement/viral load suppression, integrating the quantitative and qualitative data together to build on the strengths of both data sources to learn new insights. More specifically, analysts created a themes-by-statistics display on qualitative data related to drug use experiences, including those that discuss social relationships as they relate to use. Cases were organized by viral load status (non-suppressed and suppressed). Further, analysts added to the themes-by-statistics display a variable capturing the proportion of nominated network members who used illicit drugs. The results from these two methods of data integration were then compared to inform the interpretation of results.

## Results

### Description of the sample

As presented in Table 1, participants ranged in age from 19 to 28 years (mean = 25 years; *SD* = 2 years). They had been diagnosed with HIV for an average of 4 years (*SD* = 3 years). Almost all (96%) were assigned male sex at birth, and the majority were cisgender (i.e., not transgender; 66%) and identified as gay/homosexual (61%). Most were Latino/Hispanic (59%). A third (33%) engaged in activities in Spanish because Spanish was their primary or only language. Insufficient income was common (82%). The mean ACES-R score was 7 (*SD* = 4). HIV viral load on a log_10_ scale ranged from 2.30 to 6.13 copies/mL. Regarding the primary outcomes: a total of 72% were well-engaged in HIV care and 19% did not evidence HIV viral suppression at enrollment (data not on Table 1). Other demographic characteristics are provided in Table 1.

**Table 1:** Sociodemographic and background characteristics (M, SD, or % [N])

	Overall (*N* = 271)	Suppressed (*N* = 219)	Not suppressed (*N* = 52)
Age (years), M, SD	25.16 (2.38)	25.16 (2.37)	25.13 (2.46)
Years since HIV diagnosis, M, SD	3.90 (2.86)	3.81 (2.88)	4.27 (2.80)
*Sex assigned at birth*			
Male	95.6 (259)	95.9 (210)	94.2 (49)
Female	3.3 (9)	2.7 (6)	5.8 (3)
Intersex/other/prefer not to answer	1.1 (3)	1.3 (3)	0 (0)
Cisgender gender identity	66.1 (179)	64.8 (142)	71.2 (37)
Gay/homosexual sexual orientation	60.9 (165)	63.5 (139)	50.0 (26)
*Race/Ethnicity*			
Hispanic or Latine	58.7 (159)	59.8 (131)	53.8 (28)
Non-Hispanic African American/Black or Multiracial	41.3 (112)	40.2 (88)	46.2 (24)
Born outside US or Puerto Rico	49.1 (133)	51.1 (112)	40.4 (21)
Study Activities Conducted in Spanish	33.2 (90)	36.5 (80)	19.2 (10)
*Immigration and citizenship status*			
US citizen	53.5 (145)	50.7 (111)	65.4 (34)
Refugee, asylum, temporary protected immigrant status	35.1 (95)	37.9 (83)	23.1 (12)
Undocumented	6.6 (18)	6.4 (14)	7.7 (4)
Permanent resident/Green card	3.7 (10)	3.7 (8)	3.8 (2)
Other (tourist visa, work visa)	0.7 (2)	0.9 (2)	0 (0)
*Education*			
High school graduate/equivalent or more	82.3 (223)	84.5 (185)	73.1 (38)
Less than high school	17.7 (48)	15.5 (34)	26.9 (14)
Insufficient Income	81.9 (222)	81.3 (178)	84.6 (44)
ACES-R score (range 0–14), M, SD	7.19 (3.72)	7.28 (3.63)	6.77 (4.12)

Table 2 describes the social interaction processes for the sample as a whole and the suppressed and non-suppressed subgroups. We describe select characteristics of the whole sample here for parsimony. The average network size was 4 (range 0–5), and participants had known members for 12 years on average (*SD* = 7 years). The average proportion of members seen daily or weekly was 0.30 (*SD* = 0.32). Network members were 33 years old on average (*SD* = 8 years). Members were mainly peers (0.50, *SD* = 0.34) or family members (0.39, *SD* = 0.33). A minority (0.20, *SD* = 0.27) were diagnosed with HIV or used illicit drugs (0.35, *SD* = 0.35). The average social support score was 54 on a 0-100 scale, indicating moderate levels of support (*SD* = 24). The average satisfaction with care score was 6 on a 0–8 scale, indicating high levels of satisfaction. Engagement in care was also favorable (mean = 24 on a 0–32 scale, *SD* = 7), as was trust in the provider (31 on a 0–44 scale, *SD* = 9). Although we did not carry out tests of significance, data suggest those with suppressed HIV viral load may have a lower proportion of network members who used illicit drugs compared to those with non-suppressed HIV viral load.

**Table 2:** Social interaction processes (M, SD or % [N])

	Overall (*N* = 271)	Suppressed (*N* = 219)	Not Suppressed (*N* = 52)
*Characteristics of social network members*			
Size of network (range 0–5)	3.99 (1.30)	3.95 (1.32)	4.17 (1.23)
Years known	11.89 (6.69)	12.08 (6.81)	11.05 (6.16)
Proportion of network members seen daily or weekly	0.30 (0.32)	0.31 (0.33)	0.25 (0.25)
Age of network members (years)	32.97 (8.43)	33.00 (8.66)	32.82 (7.41)
Proportion of network members - peer (friend)	0.50 (0.34)	0.50 (0.34)	0.46 (0.33)
Proportion of network members - family member	0.39 (0.33)	0.38 (0.33)	0.40 (0.32)
Any Romantic Partner in Network	25.5% (65)	23.2% (48)	35.4% (17)
Proportion of network members - cisgender male	0.40 (0.28)	0.38 (0.28)	0.48 (0.27)
Proportion of network members – sexual/gender minority	0.53 (0.33)	0.52 (0.33)	0.57 (0.34)
Proportion of network members - African American/Black (non-Hispanic)	0.39 (0.43)	0.38 (0.43)	0.44 (0.41)
Proportion of network members - Latino/Hispanic	0.55 (0.43)	0.57 (0.44)	0.47 (0.41)
Proportion of network members - Other race/ethnicity	0.06 (0.14)	0.05 (0.13)	0.09 (0.16)
Proportion of network members diagnosed with HIV	0.20 (0.27)	0.20 (0.28)	0.19 (0.24)
If diagnosed with HIV, takes HIV medication most of the time	0.90 (0.28)	0.92 (0.25)	0.82 (0.38)
Proportion of network members who use illicit drugs	0.35 (0.35)	0.32 (0.34)	0.49 (0.37)
Proportion of network members - currently in school or college	0.19 (0.25)	0.20 (0.26)	0.15 (0.21)
Proportion of network members - currently working on-the-books	0.54 (0.34)	0.54 (0.34)	0.51 (0.33)
Proportion of network members in school or employed on the books	0.60 (0.35)	0.61 (0.35)	0.56 (0.35)
*Intimate partner violence (IPV)*,* %*			
Lifetime intimate partner violence victim	44.6 (121)	44.3 (97)	46.2 (24)
Past six months intimate partner violence victim	10.5 (28)	8.8 (19)	17.6 (9)
Lifetime intimate partner violence perpetrator	31.7 (86)	31.5 (69)	32.7 (17)
Past six intimate partner violence perpetrator	6.4 (17)	5.6 (12)	9.6 (5)
IPV victim or perpetrator in the past six months	11.3 (30)	9.8 (21)	17.6 (9)
Social Support Total Score (range 0-100)	54.14 (24.20)	54.19 (23.87)	53.91 (25.83)
HIV Care Satisfaction Total Score (range 0–8)	5.61 (2.23)	5.75 (2.11)	4.93 (2.65)
HIV Care Engagement Total Score (range 0–32)	25.04 (6.64)	25.22 (6.17)	24.17 (8.59)
Provider Trust Total Score (range 0–44)	31.22 (8.60)	31.77 (8.30)	28.86 (9.53)

Table 3 shows unadjusted (i.e., bivariate) and adjusted (i.e., multivariate) odds ratios relating social interaction processes and background variables to HIV care engagement. In the multivariate model, adverse childhood experiences (ACES) decreased the odds of HIV care engagement (OR = 0.90; *p* = 0.025). When considered alone in bivariate models, completing study activities in Spanish (OR = 3.09; *p* = 0.001) was associated with increased odds of being well-engaged in HIV care, while years living with HIV (OR = 0.90; *p* = 0.029), non-Hispanic African American/Black race/ethnicity (OR = 0.51; *p* = 0.014), adverse childhood experiences (OR = 0.91; *p* = 0.011), and proportion of network members who use drugs (OR = 0.38; *p* = 0.014) were associated with decreased odds of being well-engaged in HIV care.

**Table 3:** Bivariate and multivariate predictors of being well-engaged in HIV care

	Bivariate			Multivariate		
	OR	OR 95% CI	*p* value	aOR	aOR 95% CI	*p* value
Age	0.93	0.83–1.04	0.208	0.94	0.82–1.08	0.418
**Years Living with HIV**	0.90	0.82–0.99	*0.029*	0.94	0.84–1.05	0.268
Cisgender gender identity	0.75	0.42–1.33	0.322	0.68	0.34–1.38	0.286
Gay sexual orientation	1.54	0.90–2.64	0.116	1.31	0.68–2.51	0.424
**African American/Black Race (Non-Hispanic)**	0.51	0.30–0.87	*0.014*	0.87	0.41–1.82	0.711
**Study Activities in Spanish**	3.09	1.59–5.99	*0.001*	2.41	0.89–6.62	0.085
Education less than high school	0.91	0.46–1.82	0.799	0.99	0.44–2.23	0.985
Insufficient Income	0.53	0.24–1.16	0.112	0.53	0.22–1.30	0.168
**ACES-R Sum**	0.91	0.84–0.98	*0.011*	0.90	0.84–0.99	*0.025*
Number of network members listed	1.06	0.86–1.30	0.604	1.08	0.86–1.36	0.481
Proportion of network members who are SGM	1.10	0.49–2.48	0.819	0.88	0.30–2.56	0.806
Any romantic partner among network members	0.70	0.38–1.29	0.248	0.72	0.34–1.51	0.376
**Proportion of network members who use illicit drugs**	0.38	0.18–0.82	*0.014*	0.66	0.24–1.82	0.420
Proportion of network members diagnosed with HIV	1.52	0.49–4.74	0.465	1.36	0.30–6.11	0.681
Proportion of network members in school or employed on the books	0.97	0.44–2.14	0.941	1.07	0.41–2.77	0.891
IPV victim or perpetrator in the past six months	0.72	0.32–1.64	0.431	1.13	0.44–2.92	0.803
Social Support Scale	1.00	0.99–1.02	0.395	1.00	0.99–1.01	0.984
Satisfaction with HIV Care Scale	1.12	0.99–1.26	0.062	1.07	0.88–1.31	0.494
HIV Care Engagement Scale	1.03	0.99–1.07	0.093	1.01	0.94–1.07	0.881
Trust in Provider Scale	1.03	0.99–1.06	0.105	1.01	0.96–1.06	0.688

Table 4 shows unadjusted (i.e., bivariate) and adjusted (i.e., multivariate) odds ratios relating social interaction processes and background variables to HIV viral load suppression. While none of the variables had a significant association with the odds of suppression in the multivariate model, when considered alone, study activities in Spanish (OR = 2.42; *p* = 0.020), satisfaction with HIV care (OR = 1.18; *p* = 0.016), and trust in provider (OR = 1.04; *p* = 0.045) were associated with increased odds of HIV viral suppression and proportion of network members who use drugs (OR = 0.32; *p* = 0.009) was associated with decreased odds of suppression.

**Table 4:** Bivariate and multivariate predictors of HIV viral suppression

	Bivariate			Multivariate		
	OR	OR 95% CI	*p* value	aOR	aOR 95% CI	*p* value
Age	1.01	0.89–1.14	0.935	1.00	0.85–1.17	0.973
Years Living with HIV	0.95	0.85–1.05	0.302	0.94	0.84–1.07	0.398
Cisgender gender identity	0.75	0.39–1.45	0.388	0.61	0.28–1.36	0.231
Gay sexual orientation	1.74	0.94–3.20	0.076	1.48	0.71–3.10	0.291
Non-Hispanic Black Race/Ethnicity	0.78	0.43–1.44	0.432	1.30	0.57–2.97	0.532
**Study Activities in Spanish**	2.42	1.15–5.08	*0.020*	2.12	0.67–6.62	0.200
Education less than high school	0.50	0.24–1.02	0.056	0.57	0.24–1.31	0.186
Insufficient Income	0.79	0.35–1.80	0.575	0.68	0.26–1.77	0.425
ACES Sum	1.03	0.95–1.12	0.404	1.05	0.96–1.16	0.247
Number of network members listed	0.91	0.71–1.17	0.473	0.93	0.70–1.23	0.636
Proportion of network members who are SGM	0.68	0.26–1.76	0.423	0.59	0.17–2.08	0.415
Any romantic partner among network members	0.58	0.30–1.13	0.110	0.77	0.35–1.68	0.514
**Proportion of network members who use drugs**	0.32	0.14–0.75	*0.009*	0.46	0.15–1.48	0.190
Proportion of network members diagnosed with HIV	1.09	0.34–3.48	0.888	1.28	0.27–6.05	0.750
Proportion of network members in school or employed on the books	1.46	0.61–3.48	0.397	1.65	0.55–4.95	0.372
IPV victim or perpetrator in the past six months	0.51	0.22–1.19	0.117	0.79	0.29–2.12	0.633
Social Support Scale	1.00	0.99–1.01	0.943	1.00	0.98–1.01	0.839
**Satisfaction with HIV Care Scale**	1.18	1.03–1.35	*0.016*	1.15	0.92–1.43	0.219
HIV Care Engagement Scale	1.03	0.98–1.08	0.198	0.97	0.90–1.05	0.459
**Trust in Provider Scale**	1.04	1.00–1.07	*0.045*	1.03	0.97–1.09	0.381

### Qualitative results

#### Overview

**Fig. 2:**
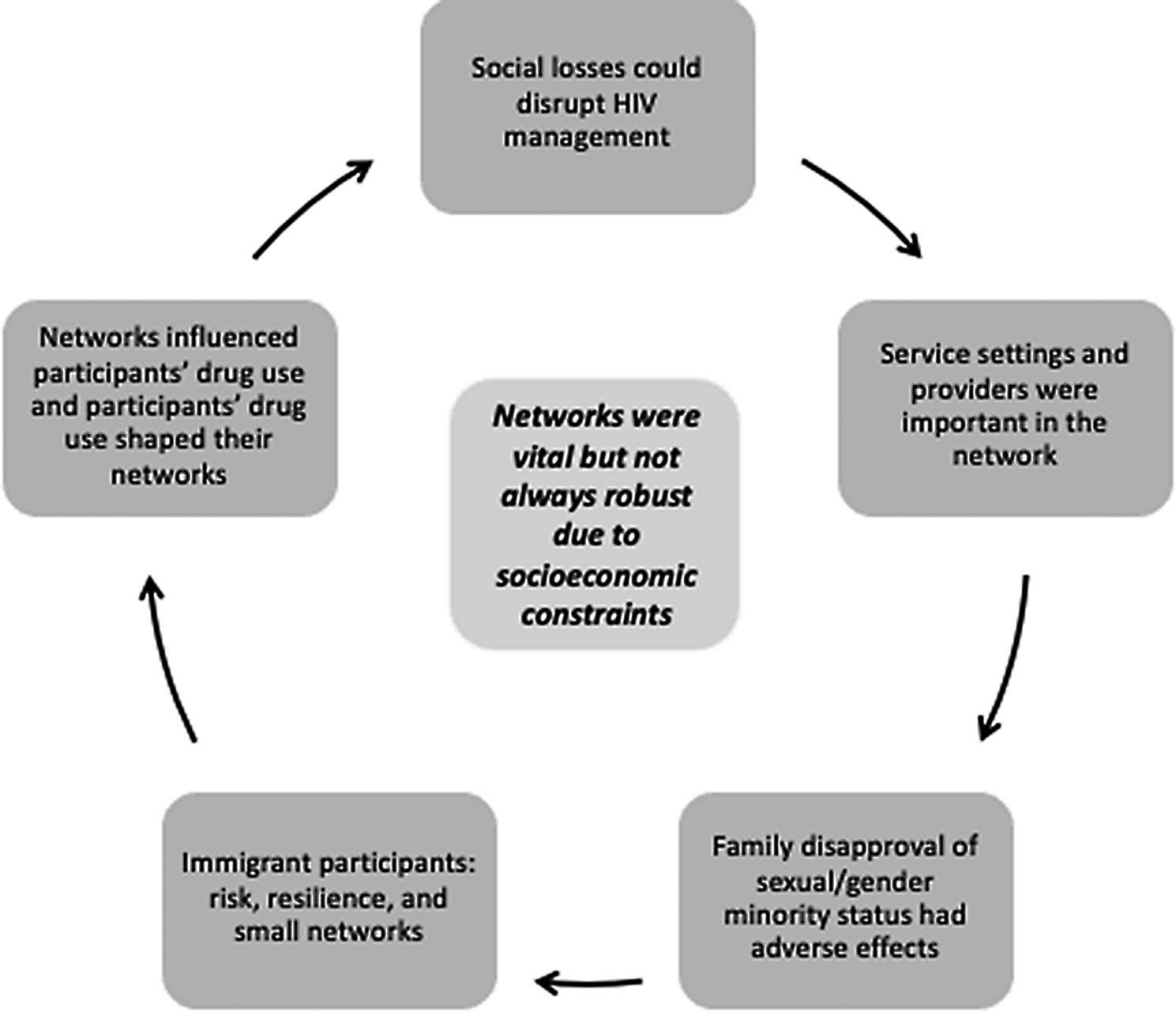
Themes identified in the qualitative analysis

Participants’ social networks were vital to their well-being, and these networks deeply influenced their lived experiences, including regarding HIV management. Participants had complex emotional, practical, and social needs, given their status as emerging adults living with HIV, in the context of racial/ethnic and sexual/gender minority status and serious financial constraints. Participants evidenced resilience, including expertise in HIV management, but their external circumstances, such as employment, finances, and housing, were, by and large, precarious. Participants were either in the early stages of their professional careers or unemployed, and financial strains and difficulties were the norm. Further, their networks were largely made up of individuals also disproportionately affected by larger socioeconomic factors and strains associated with low income and various minoritized experiences (e.g., related to race/ethnicity and sexual/gender minority status, and immigration). Thus, networks were not always secure, stable, or robust. Within this context of socioeconomic insufficiency among participants and their social networks, we identified five main inter-related themes (Fig. 2): (1) social losses could be disruptive to the network and HIV management; (2) service settings and providers were important members of the social network; (3) family disapproval of sexual/gender minority status had adverse effects; (4) immigrant participants experienced a unique set of risk and protective factors, including being highly reliant on service settings; and (5) networks influenced participants’ drug use patterns and participants’ drug use patterns shaped their networks. This latter theme (#5) was addressed both in the qualitative content and text-by-statistics analyses. Both the importance and precariousness of social networks are reflected in each of these themes. Participants are described below using pseudonyms, and some identifying details have been changed or removed to protect confidentiality. We use the participant’s preferred pronoun in each description.

### Social losses could disrupt HIV management

Participants experienced their support networks as vital in helping them meet emotional and resource needs and with HIV-related concerns, and they detailed the various ways their network members cared for them. Networks were comprised of family-of-origin, chosen family, friends, and intimate partners, as is typical of this age group, and also included health care and social service providers within service settings. Peers were vital to participants’ knowledge of and ability to access resources, both HIV and non-HIV related services. Network members commonly assisted participants with HIV medication management. Chosen family and friends commonly motivated participants to engage in HIV care, sometimes encouraging them to move to other geographical locations for better HIV care opportunities, and also offered housing and financial assistance, to the extent possible. However, we found that losing an important social network member, whether because of a breech in the relationship or a death, could have severe negative effects on participants’ motivation and abilities to manage HIV care and take medication, often because losses triggered mental health distress and/or heavy substance use. This was the case for Darrell, a Black man in his late twenties, who experienced the loss of his mother a few years after his HIV diagnosis, which, in turn, triggered mental health symptoms, and this interfered with engagement in HIV care:

So, my mom, her birthday was October 18th. We had a [car] wreck. […] She ended up having a heart attack and we had to take her off life support in November. I was, excuse my French, fuck it all. Like I really wasn’t focusing on my [HIV] treatment. That wasn’t my top priority at that time. […] [As far as HIV medication it was] off and on. Not like I was supposed to, but every blue moon I would [take it]. […] To be honest. This was one time I was really depressed and suicidal, so. I basically, like, gave up, really.

Yet, Darrell, despite going down a “dark road” for a long period of time, characterized by depression, alcohol and drug use at elevated levels, and avoidance of HIV care, decided to make a change in his life.

I made a vow to myself. […] It was before Christmas. I said, with my next paycheck, I’m leaving, and I’m not coming back. […] I said wherever my last paycheck takes me, I’m going to go. So. I ended up [in a large northeastern city]. […] I think I wanted it to be where I didn’t have no one. So, I can experience, you know. What it’s like to be an adult. […] As soon as I got here, I got all that taken care of or whatnot [linkage to HIV care].

In addition to underscoring the adverse effects that social network losses might have, Darrell’s experiences also highlight the central role that HIV care and social service settings play in the lives of this population of emerging adults, along with a growing sense of autonomy over his health care, a hallmark of adulthood.

David, a Black man in his late twenties whose support network was primarily made up of friends, similarly described the adverse impact of a loved one’s death on his mental health and HIV medication adherence, along with the importance social network members can have in supporting well-being and re-engagement in HIV care.

[I took HIV medication] when I first got diagnosed. Then I stopped. Because I went through a dark period when my father passed. I just checked out of life for a second. And then I checked back in. […] So, I started back and everything. […] My best friend, she helped me. […] She moved me from [a large southern city] to [a large northeastern city], and just helped me become myself again, because I was literally in the house, in the bed, not doing nothing, not talking to anyone. She literally flew down to [the large southern city] and came to my house, beat down my door, and said, “Uh-uh, we’re not gonna do this.”

Thus, losses of network members commonly triggered lapses in HIV management, but networks and HIV care settings could also help participants re-commit to HIV care and medication.

### Service settings and providers were important members of the social network

Service settings and providers, including HIV care services, were generally important aspects of participants’ social networks. Most of the time, but not always, they were experienced as important and positive sources of tangible and social support, including regarding HIV management, but also more generally. As noted above, the robustness of social networks was often precarious because of strained socioeconomic circumstances among members. Similarly, HIV service settings and providers were dependent in part on philanthropic and/or government funding, and therefore changes in donations or grants could negatively affect services. We describe both positive and negative aspects of service settings and providers in this section. Settings and providers generally assisted with HIV medication initiation and management, and other needed resources (e.g., insurance, housing). Further, because of their deep knowledge of living with HIV as an emerging adult, and the specific concerns that AABL and lesbian, gay, bisexual, transgender, and queer (LGBTQ) persons face, they also provided participants with sorely-needed emotional and social support. For example, Andre, a Black man in his mid-twenties, described his support network as being made up of his mother, partner, doctor, and a local LGBTQ organization. He valued the treatment he received at the LGBTQ agency he attended:

The [local] area may be bad, but inside the workers actually try hard to, you know, do their best and make us feel comfortable, to feel loved, feel that we have a place to go to because our parents don’t accept us, who we are, or what we’re trying to be.

Thus, participants valued settings and providers competent in HIV care, as well as those expert in and committed to the psychosocial issues that LGBTQ persons face, both generally and regarding HIV management. It was common for participants to experience strong, trusting, and long-standing relationships with their health care providers. For example, Terrence, a Black man in his late twenties, whose network members included family members, a chosen sister, friends, and his HIV care provider, stated:

She [my provider] said I can call her anytime. It’s actually good. I talk to her about anything when I go. Normally, if I have any questions, I normally save them for when I have to see her. But we have a good relationship. It’s easy to talk to her.

However, as noted above, HIV care settings commonly experienced constrained resources. Participants noted that care settings varied in their abilities to provide personal and convenient services. Further, the transition from pediatric/adolescent to adult services was often challenging, since adult settings provided less structure and guidance than pediatric clinics. Thus, adult care settings where providers had large caseloads could be challenging to navigate. For example, Bryan, a Black non-binary individual in their late twenties, who recently relocated to a large urban area from another mid-sized city in the northeast US, discussed how their network included mainly friends also living with HIV. They described the experience of receiving care in a sizable adult clinic in a large urban area, in comparison to other types of settings:

I kind of miss just maybe a more hands-on [approach], but just [the large urban area] is huge. My doctor sees a gazillion people a day, so yeah. I had more of a support system at [the previous care setting], you see everybody [different types of providers] on one visit. But here, it’s like, oh, you’re fine, we’ll see you in three months. […] I loved my adolescent care. […] [It] kind of shaped who I am today and how I look after myself. […] They were very on top of me and how I did things. Specifically, my own social worker […] we kind of grew a close bond.

Providers were necessary in the lives of participants, and while most were considered constructive members of the network, it was not uncommon for providers to be described as lacking in support and positive interactions. The reasons for such dissatisfaction included HIV stigma, “surveillance” of their HIV medication use (described below), and poor treatment by clinic staff perceived as transphobic. Carmen, a Black transgender woman in her late 20s, missed HIV care appointments because of poor treatment by staff in the setting, which, she noted, was in part a reaction to Carmen’s transgender identity:

I would miss certain appointments just because of the fact that I’m discriminated against in ways to where a guy and these ladies pushed the needle in my arm all wrong and stuff, and they just make me so uncomfortable. The ladies don’t like me being at all with the trans situation ‘cause they always having like these negative comments when I come in and then she shoves it [the needle] into my arm, and they always have to fix it till the blood can continuously come out ‘cause I have small veins. So, they have to continuously fixing it and then shoving it in and pulling it out. And it just, it’s so uncomfortable. It makes me not want to go. […] Also, the fact that we have to go through the, we call it the ugly stages, which is just feeling like we look like our old selves. […] And you could be beautiful [as you transition] and they’ll still make faces at you to make you think you’re ugly.

As noted above, some participants experienced settings and providers as surveilling, rather than supporting, their HIV medication-taking patterns, and this contributed to dissatisfaction with HIV care. Surveillance might include being required to attend health care appointments and receive HIV viral load testing monthly in order to get prescriptions, rather than on a more liberal schedule. This type of close supervision of HIV medication use was perceived as infantilizing, as Cedric, a Black gay man in his mid-twenties, noted:

They want to give me labs every month so that they can make sure that I’m taking the pills. […] That’s annoying. If you got [health care setting’s] viewpoint of me, they probably wouldn’t think that I was an adult.

Thus, service settings, including LGBTQ-specific venues, and the medical and social service providers located within them, were important aspects of participants’ social networks, although the transition from adolescent to adult care settings could be difficult. Relationships with settings and providers were generally positive, but not always. Transgender participants in particular experienced multiple layers of stress and, in some cases, discrimination.

### Family disapproval of sexual/gender minority status

Parental disapproval of sexual/gender minority status was common, mainly in the past, and ranged from outright rejection to eventual partial or begrudging acceptance. Full acceptance of ones’ identity was not the norm, but it was possible, most commonly from mothers. Participants did not always disclose their HIV diagnosis to their family-of-origin, which limited the support for HIV management that families might provide. But, not disclosing their HIV status to family also protected them from the family’s negative reactions or distress (“it would kill them if I told them”) and/or stigma. It was also common for participants to hide their sexual minority status from family, mainly fathers. Negative views on HIV, homosexuality, or non-cisgender gender identity were not the norm in networks, but when present, for example, in the family-of-origin, they could have serious adverse effects on participants. It was more common for fathers than mothers to have negative views on homosexuality or gender-nonconforming gender identity. Some participants had no communication with family members because of sexual orientation/gender-based stigma. Others maintained their relationships with parents who held negative views on queer sexuality or gender-nonconforming gender identity, but these relationships were strained. Martin, a Black man in his early twenties whose network included a LGBTQ support group, family, and friends, explained that his distance from his mother was a result of “homophobia” and physical abuse:

She’s kind of like very homophobic, and then she [was] kind of physically abusive. Like [when] I was 20, I guess, that time she hit me and didn’t want to stop. I didn’t want to live in that environment. So that’s why we don’t have that physical relationship.

Thus, family disapproval of sexual/gender minority and/or HIV status could fracture or end family relationships. For instance, Andre, introduced earlier, communicated with his mother but was estranged from his stepfather because of his intolerant views on HIV and queer sexuality:

My stepfather still doesn’t know [about my HIV diagnosis or gay sexual orientation]. I talk to my mother. But my step-pop and me never will get along. As much as he can try to hide it, he does not like people who are HIV-positive, or who are gay. He can hate, but I got love.

Similarly, Rosa, a Latine transwoman in her mid-twenties, described having a relationship with her mother but no communication with her father and siblings:

I have my parents, and brothers. [I communicate] with my mom more than anyone. She does accept me as I am but well, no, not my brother. Neither does my father. I have two brothers here in the United States. And no one wanted to support me, no one wanted to help me when I needed it because they don’t accept me as I am.

Rosa provided another example of fathers being more likely to reject or stigmatize their children than other family members, and also highlighted that men overall are more likely than women to lack tolerance of sexual/gender minority status and HIV. Participants compensated for these losses by building up and relying on chosen family, as Sophia, a biracial transwoman in her early twenties, described:

I definitely have my chosen family here. I was just telling my partner earlier that my [chosen] family, my friends, we go in for each other. My sister, it makes me, literally, cry. I literally teared up this morning thinking about how hard she goes for me. And what a beautiful relationship we have to go through this experience with someone who is also trans, but also Black and trans.

Yet, relationships with family-of-origin could improve over time, with benefits to well-being and HIV management, as Elijah, a Latine man in his early 20s, discussed:

[Regarding his family of origin and their reaction to learning about the HIV diagnosis], it’s starting to get better. […] They definitely do [support me]. They definitely ask if I’m, like, taking my medication. So, they definitely support and worry about me. […] [I take medication daily because of] my family. The fact that they care. They ask every day, daily, if I’m taking it. And how am I feeling, and my brother who works in the hospital, he knows about stuff like that. So, he’s definitely on me about it. […] I think that my friends love me deeply and they always like, trying to ask me how I am, if I’m okay. I know not only because [I am] HIV-positive, but about everything else, you know. Because there’s much, much more in our lives than just this little part of it [the HIV diagnosis].

These results highlight that HIV management, a complex and chronic challenge for emerging adults, is largely dependent on, or at least sensitive to, the quality of support within social networks.

### Immigrant youth presented unique issues

Immigrant participants, all from Latin American countries (namely, the Caribbean and Central and South America) and the majority monolingual Spanish speakers, tended to describe having fewer social network members and other resources in the US compared to non-immigrant participants, both locally and in their home country. It was common for participants to have conflict with or end relationships with family members because of their decision to immigrate and/or due to conflict related to participants’ sexual minority identity. The hope for better economic opportunities and/or to escape LGBTQ discrimination and violence were major reasons participants noted for leaving the home country. Those who were diagnosed with HIV in Latin America also commonly sought to leave as a means of accessing higher-quality HIV care and HIV medication. Thus, service settings, including those with Spanish-speaking staff, and other Spanish-speaking peers living with HIV, were particularly important to these participants as they sought access to basic resources in the US. For example, Javier, a Latine man in his late twenties, came to the US seeking a higher income for his work as a social media manager compared to the wages he was able to earn in his home country. Now living in a large metropolitan area in the northeastern US, he actively participated in and relied on the local LGBTQ services ecosystem to meet his needs:

I’m going to go to Project [name redacted]. […] There are many groups here. The first group is at a LGBTQ Center. They have meetings. They ask you what state [condition] you are in. You say, “I’m looking for a shelter, I’m looking for something, I don’t have money.” You start to communicate with the LGBTQ community. There is always a big network of support. There are many new LGBTQ organizations. They help you with clothes, food, and they give you a bonus to buy food.

Immigrant participants also relied on their networks, particularly service agencies, during their migration journeys and specifically for linkage to HIV care once arriving in the US. Lionel, a Latine man in his early twenties, migrated from his home country to a neighboring country (where he was diagnosed with HIV) after receiving threats of sexual violence from gangs because of his sexual orientation. He then moved to Mexico with the intention of migrating to the US for higher-quality HIV care. Lionel described an LGBTQ and HIV-serving organization in Mexico as being especially supportive in his migration journey:

[I got a sponsor in the US] through the hostel [in Mexico]. […] They help migrants and people who are positive like us. […] When I arrived [in a large US city], they put me in [HIV care], the day I arrived, within three days. They put me in a clinic called [name redacted]. The woman [my sponsor] who picked me up in [the large US city], she works in the clinic.

Although networks were generally effective in supporting immigrant participants and introducing them to needed tangible resources, Andres, introduced above, demonstrated how one’s immigration status could also disconnect individuals from sources of support and impede HIV management. Andres was applying for asylum status in the US and his Merits Hearing, where his case would be presented to an immigration judge, was scheduled for several months in the future. Yet, he could not apply for HIV social services in the US until after this hearing. Without services and public benefits, and while he waited for his hearing, he lived in a shelter and depended on inconsistent work in the informal economy for his survival. Andres described having a heart attack due to the stress of his situation:

When I told you that I am recovering on Sunday, I had a heart attack. As of yesterday, I was discharged from the hospital, and I am convalescing. I’m taking care of myself, I have a patch on my chest and I’m being monitored for the same thing, for stress, crisis, anxiety, and problems. Because my dad died recently, and I haven’t been able to cope with my grief and I was really upset with that. That has given me a lot, a lot, a lot of depression. And apart from that, with the problem of migration, with the problem of [the local HIV services administration] that has not given me the support I need, is that I am without a job. So, all of that has given me a lot of pressure and that’s what has happened to me today.

These findings highlight the critical role federal benefits/entitlements, the HIV services network, and LGBTQ community-based organizations played in the lives of immigrant emerging adults living with HIV, who typically entered the US with minimal social support, financial resources, English language skills, and knowledge of how to access services. However, for many, HIV care and medications were interrupted during the migration journey, and when in the US, federal benefits, housing, employment, and HIV management resources were delayed due to lengthy or delayed legal proceedings.

### Networks influenced participants’ drug use patterns and participants’ drug use patterns shaped their networks

Network members supported participants generally with material and emotional needs, and also commonly supported HIV management directly by reminding them to take HIV medication. They also intervened when conditions, such as participants’ heavy drug use or depression, interfered with medication taking, as we describe throughout this section. Network members were generally described as positive influences, but drug use could shape or change networks in ways that were ultimately detrimental to participants. Participants were commonly introduced to drug use by network members, and these members could be important in providing them with drugs and using drugs with them. Participants described alcohol and drug use as a means of coping with stress, including the stress of living with HIV. In some cases, participants were exposed to drug use in their family of origin. Some participants described avoiding substance use in response to seeing family members struggle with addiction. Later in their lives, however, drug use was common, or even normative, including in LGBTQ social venues (“It is literally everywhere”). HIV and health were reasons participants gave for avoiding drugs, at least at times, but the high prevalence of drug use in their social networks could make abstinence from substances difficult.

Commonly, participants used drugs in ways that were social and non-hazardous (“it’s mostly like sometimes to sleep or when I’m with friends, but I don’t do it a lot either”). In other cases, however, drug use at hazardous or problem levels was encouraged and facilitated by, or even coerced by, network members, such as romantic partners. For example, Cedric, a Black man in his mid-20s, described being introduced to drugs in a “transactional situation” with a “sugar daddy” at the age of 14. He noted:

He’s the reason why I am on hard drugs. And I started doing that shit then. So now, like I explained to him that, like, I didn’t ask for this problem. I wish I would have not done it. […] So, it’s just like, I kind of don’t know how to do anything else other than, like, binge hard drugs. And, like, when I’m not bingeing hard drugs, I’m like, bingeing sleep um, and mother-son arguments.

Cedric described himself as having a small social network as a younger person, and currently had support but focused mainly on maintaining those relationships that provided easier access to drugs, including transactional relationships where companionship or sex were exchanged for drugs. He had recently returned from drug treatment and obtained a job, and intended to change his drug use patterns, with the assistance of federal nutrition and income support programs, and HIV social services and housing organizations.

Thus, networks influenced participants’ drug use, and further, drug use could affect the network size and composition, whether it was the participant’s use and/or use among network members. In particular, heavy drug use strained relationships with and among network members. Participants would avoid friends and family during times of heavy use to avoid stigma (“I was hiding it from everyone that I was using again because I had a relapse”). Drug use within romantic partnerships could complicate the relationship, as well as efforts to reduce or eliminate use. Sophia, introduced above, recounted how her use of methamphetamine affected her relationships with family members and the number of people she could depend on for help:

My mom had no idea I was on meth. I hid it from her. Her husband knew. One time, he walked in my house. He was like, “You’re high on meth.” I was like, “Yeah.” He was like, “Well, I’m going to take your mom home and not let her come in [your] house.” He, my sister and my roommate knew, but those are the only people that were coming around me at that point. I was really alone most of the time.

Thus, drug use could change the size and composition of networks. For example, participants’ efforts to stop drug use could shrink their networks, such as when efforts to limit, change, or end their alcohol or drug use patterns required eliminating time with friends who used substances (“We don’t be hanging out with nobody because before, we used to do a lot of drinking and shit. So right now, we’re clean. We’re trynna keep it like that”). During times of heavy substance use, participants prioritized network members who could provide access to a regular supply of drugs and/or who they would use drugs with, and at the same time reduced contact with those who could not or did not support substance use needs. Cedric, introduced above, explained why he ended a relationship with a friend:

He’s not really my friend anymore. I get really demanding when I want to get high. He always has the drugs I want to do, but he wants to tell me how to use them…because he cares about me. […] He’s just like, I’m not going to get you high because I don’t want you to slam [inject drugs, usually methamphetamine, intravenously]”. […] Because all it takes is one air embolism. […] I get so angry. […] I really hate when people like [confine me]. It’s in my nature to abolish them. If I can’t, then I get real bent out of shape.

### Data integration

Table 5 provides a comparison of the quantitative and qualitative results using the joint display framework, and notes if results were congruent, discrepant, and if they raise additional research questions. We summarize the findings briefly here. Overall, the qualitative results added context, depth, and meaning to the quantitative results. For example, quantitative data indicated a relationship between drug use among network members and challenges to HIV management. The qualitative data added insights into the mechanisms by which social interaction processes operated to influence well-being generally, and substance use and HIV management specifically.

Results from a text-by-statistics analysis suggested a higher proportion of participants in the non-suppressed HIV viral load subgroup reported current drug use in the qualitative data, when compared to those in the suppressed subgroup (60% vs. 26%). Examining data on social networks and relationships as it related to alcohol and drug use, those who were HIV virally suppressed rarely discussed drug use in the context of social relationships. Further, when they did discuss substance use and social relationships, they discussed how use led to new social relationships (e.g., with a professional counselor), was used in a way they considered destructive (being ‘invited’ to use with strangers), and that social network members (e.g., romantic partners) assisted them in stopping use. Importantly, in this group, some participant responses implied they used drugs alone and/or used drugs to cope.

In contrast, in the non-suppressed subgroup, data suggested that participants used drugs and alcohol with members of their social network. Further, participants reported the loss of life of their loved ones related to substance use (“A lot of my friends and family, they didn’t make it through addiction”). More specifically, participants discussed doing cocaine with friends, “growing up around” drugs, and romantic partners both introducing them to drugs and in one case repeatedly supplying them with drugs. These qualitative data further support the quantitative finding that the proportion of network members who use drugs reduces the odds of being well-engaged in HIV care and being virally suppressed, while also providing examples of drug use experiences with members of the participants’ social networks. Relatedly, those who reported substance use in the suppressed viral load group (*n* = 10) all reported using marijuana and alcohol, with the exception of one participant who reported more extensive drug use. In the non-suppressed subgroup, on the other hand, of those who reported using drugs (*n* = 12), 42% reported using drugs with a higher risk of addiction or adverse consequences, such methamphetamine and cocaine, as well as mushrooms/psilocybin. These participants also discussed selling drugs and perceiving themselves as having addictive behaviors, “It was real crackhead energy. It was like, ‘All right, guys. I’m going to the store’, but really I’m behind the park smoking meth.”

Finally, we examined across the two subgroups if there was an association between reporting network members’ use of illicit drugs and the participant’s use/non-use of substances. Data suggested that in 64% of the cases there was a “match” related to substance use/non-use. Our team operationalized a match as either (1) participants who reported that half or more of their social network members used drugs, the participant also described using drugs, or (2) participants who reported fewer than half of their social network members used drugs, participants did not describe using drugs themselves. While exploratory in nature, these results are in line with social support literature that suggests substance use behavior is influenced by the use/non-use behavior of important social network members.

**Table 5:** Integration of data sources using a joint display to integrate and interpret findings

Quantitative finding	Qualitative finding	Comments and Interpretation
Drug use was common among network members and the proportion of network members who use drugs was close to 50% among those with non-suppressed HIV viral load	Qualitative data yielded insights into the dynamics of substance use in networks and how substance use by the participant and/or network members could shape networks. There appeared to be a synergistic relationship between participants’ substance use and that of their network members.	Qualitative and quantitative data were consistent and qualitative results added context to quantitative results regarding the importance of drug use in social networks.
The higher the proportion of network members who use drugs reduced the odds of being well-engaged in HIV care and HIV viral suppression	Participants did not generally explicitly discuss how substance use among social network members influenced engagement along the HIV care continuum. But, discussion of substance use was more prevalent in the networks of those with non-suppressed HIV viral load compared to those with suppressed viral load.	Qualitative results extend quantitative results and also suggest the need for more research on the processes and mechanisms by which network members influence each other’s drug use and HIV management.
Other characteristics of social networks (e.g., number of network members listed, proportion diagnosed with HIV, in school or employed on the books) were not associated with HIV care engagement or HIV viral suppression	Qualitative data indicated that networks were vital to participants’ ability to function and thrive. But, participants, their social network members, and to some extent, HIV care settings, were located in socioeconomically strained contexts. This affected the ability of networks to provide practical and tangible support consistently.	Characteristics of the network may limit our ability to make inferences (restricted range, low proportion of members also living with HIV).
Those whose primary language was Spanish, all of whom were immigrants, were more likely to be well-engaged in HIV care and evidence HIV viral suppression than their non-Spanish speaking/non-immigrant peers.	Immigrant participants, who reported having smaller local networks than their non-immigrant peers, were highly reliant on service settings to meet needs, along with other immigrant peers to inform them about services. It was common for these emerging adults to come to the US and resource-rich cities within the US specifically to receive HIV care and services.	Results taken together indicate that immigrant participants commonly leave their home countries in part for better HIV management supports, and that they are indeed successful in accessing supports and engaging along the HIV care continuum, despite the very serious challenges inherent in doing so. The present study points to social networks as critical in guiding participants to services in the US, and also underscores the essential role that immigrant-friendly service settings and Spanish-speaking providers play. These findings also highlight the need to better understand why US-born participants, who do not have language, documentation, or insurance barriers, are less likely to achieve optimal HIV outcomes.
Factors associated with HIV care and HIV viral suppression other than the proportion of network members who use drugs differed in some respects. For example, satisfaction with HIV care and trust in providers was positively associated with viral suppression but not HIV care at statistically significant levels.	Not explored in the qualitative data; we focused the mixed methods aspect of the study on social network members.	Factors that promote or impede HIV care engagement and viral suppression appear to have both similarities but also differences. The two health outcomes are related, certainly, but results suggest that for precision and to support health behavior, it is useful to consider the ways they differ.

## Discussion

This study took a mixed methods approach to explore the social interaction processes that influence HIV management for AABL emerging adults living with HIV, who experience a disproportionate burden of HIV infection, barriers to engagement along the HIV care continuum, and poor HIV health outcomes [5, 8]. Results highlighted the importance of social networks in encouraging health behaviors, enhancing knowledge of resources, and supporting general well-being, consistent with the existing literature [70]. Social networks are critical for all emerging adults. AABL emerging adults living with HIV may be particularly reliant on their social networks because they must manage both milestones and tasks typical of this developmental period, but also atypical challenges, including managing a chronic and stigmatized health condition that necessitates daily medication and frequent health care appointments. Notably, the majority in this cohort showed satisfactory engagement in HIV care and HIV viral suppression, despite the difficulty of doing so in the context of this developmental period with many competing priorities.

One finding from the present study was the extent to which both participants and their social networks are located in socioeconomically strained circumstances. Participants and their network members, including the HIV care settings and providers within those settings that serve them, commonly lack sufficient resources, which strains their abilities to support each other. Yet in this context, we found social networks commonly directly and indirectly support engagement along the HIV care continuum, along with well-being generally. Overall, peers played more prominent roles in the network compared to family, as is typical of this developmental stage [71, 72]. However, in this cohort, family relationships were often strained by issues such as parental disapproval of sexual/gender minority status, family reaction to the participant’s HIV status or the participant’s desires to keep their status from them, and drug use, as has been found in past research [73, 74]. This suggests that AABL emerging adults experience stigma from their families. Regarding peers, the proportion of network members diagnosed with HIV was low, which may limit the network’s ability to effectively support HIV management. The population engages regularly in HIV care settings, where they might, in theory, create bonds with other people living with HIV. But, there may be barriers to doing so, such as stigma or a lack of structured opportunities to build connections [75]. Thus, AABL emerging adults living with HIV have both typical and atypical social network needs along with typical and atypical network challenges.

We found that social networks shape participants’ drug use, and participants’ drug use shapes their networks. For example, a higher proportion of social network members who use drugs was associated with decreased odds of being well-engaged in HIV care and with HIV viral suppression at the bivariate level. The qualitative and mixed methods findings also provide participants’ perspectives on drug use in their networks and its effects. The existing literature highlights the important role of social networks in the initiation and perpetuation of drug use, through mechanisms such as social norms, and also the network’s role in supporting harm reduction and cessation of use. Consistent with the present study, this literature suggests close family and friends can be a “mixed blessing” with respect to drug use [76].

Social network formation is a complex process in which a set of individuals simultaneously attempt to satisfy their goals under multiple, possibly conflicting, constraints [77]. Social networks evolve over time, as individuals create and deactivate social ties, driven by the shared activities and affiliations of their members, and by the similarity of individuals’ characteristics [77]. Some research has shown that persons with substance use disorders have smaller networks, and smaller networks are associated with both substance use disorder onset and persistence [78, 79]. Our study uncovers potential mechanisms that lead to smaller networks, and substance use within networks. This includes participants reducing contact with network members during times of heavy use and, at the same time, increasing contact with persons who can provide substances to them or who also use substances with them. Our findings also yielded a number of other factors that reduce network size, and these, in turn, may influence substance use patterns indirectly; namely, losses of important social network “linchpins” (e.g., mothers), being disconnected from service settings and providers, and family disapproval of sexual/gender minority status. Thus, the present study yields insights into how and why social networks may expand or contract, and the effects of these changes on well-being, including substance use.

Our study suggests that participants’ scarce financial and tangible resources place pressure on them to obtain needed drugs, and this shapes the network by increasing ties with members who can meet those needs. The present study extends a recent past paper on this cohort from our team, where we found a high prevalence of alcohol and drug use among participants, in part related to its availability in venues where participants socialized, such as LGBTQ venues. In that paper, substance use was more common among those with non-suppressed versus suppressed HIV viral load. However, while common, substance use did not typically interfere substantially with HIV management, although methamphetamine could be problematic [22]. The present study shows the extent to which social network members’ use of drugs plays a critical role in HIV management. In the qualitative analysis, participants in the present study did not explicitly link social networks or drug use in the network to HIV care or medication use. However, our studies, taken together with the existing research literature, suggest that when drug use is introduced into social networks in a context of scarce resources or other constraints or adverse events, the network may reduce in size and the proportion of those using drugs therefore increases. This smaller network may experience “competing priorities” and also encourages drug use-related norms and behaviors, and may be less effective in supporting complex health behaviors such as HIV management. These findings thus extend existing research on network approaches for understanding social influences on substance use [26].

### Implications and recommendations to advance health equity

The present study yields some insights into the development, enhancement, and/or implementation of equity-oriented interventions to bolster HIV management and well-being and prevent lapses in HIV care and medication. First, the study suggests the need for interventions at the structural level of influence. As noted above, the networks of AABL emerging adults living with HIV exist in constrained socioeconomic contexts, and experience differential access to resources. As a result, their hardiness in times of loss or change can be inadequate. Thus, interventions that address structural factors affecting networks, related to insufficient financial and other material resources, may be useful. One example of interventions to support the financial well-being of network members would be universal basic income [80, 81]. It also highlights the need for interventions at the social level of influence. Interventions to improve ties with the family, including fathers, may have potential. Indeed, some studies have suggested that fathers are “sidelined,” but that fathers are important for the well-being and development of emerging adults [82]. Peterson and colleagues examined peer support within HIV health contexts. Their results indicate that peer support is a potentially important addition to clinical care for enhancing coping skills, which, in turn, improves the psychosocial functioning of people living with HIV. They recommend assessing patient access to peer support, providing opportunities for peer support in the clinical setting, and enhancing disclosure and support-seeking skills to facilitate this potential resource [83]. The present study also highlighted that while bonds with family members are important, a family’s negative views of HIV and sexual/gender minority status, particularly among fathers, are points of intervention, since these views cause AABL emerging adults living with HIV to hide their HIV status and other disclosures from them [84, 85]. Participants may experience stigma from families, and past research shows a strong association between stigma and poor HIV management [86, 87]. Interventions to reduce HIV-related self-stigma and family stigma exist, as a review by Ma and colleagues indicates, and can be implemented in HIV care settings [87] and adapted as needed for this population. Interventions with persons experiencing hazardous substance use or mental health distress, including depression, can incorporate a focus on the social network as a risk and protective factor [26, 88, 89]. For example, it is possible to incorporate harm reduction or other substance use risk reduction approaches in network-based interventions involving friends, partners, or family. Findings also suggest points of intervention at the individual level of influence. Treatment approaches for individuals, such as cognitive-behavioral therapy and group-based treatment, can bolster skills and provide social support. Sansom-Daily and colleagues (2012) reviewed psychological interventions for emerging adults with chronic illness. They found that skills-based interventions (e.g., communication skills) delivered over multiple sessions may yield the most positive results [90]. These recommendations complement the existing body of evidence on interventions to harness the power of or to change/improve social networks [91–94]. Further, since AABL immigrant participants are highly reliant on service settings, study findings also highlight the importance of language-concordant and culturally relevant services, including bilingual/bicultural staff, and provider competence on immigrant-specific stressors such as documentation concerns, fear of deportation, or limited insurance eligibility.

### Strengths and limitations of the study

Strengths of the study include the mixed methods approach, which yielded a rich understanding of the phenomena under study and insights that would not have been generated in single-method studies, either qualitative or quantitative. Further, the diverse cohort studied here led to a better understanding of subpopulations among AABL emerging adults living with HIV that are understudied in the literature (e.g., immigrants, Spanish-speaking individuals, those not HIV virally suppressed). The study findings have implications for clinical practice and future research. The study also has limitations. First, the social factors studied here may interact with domains in the model at other levels of influence, which limits the number and types of associations found. We acknowledge that social interaction processes likely interact with other factors in the model. This paper is intended to take a first step in exploring social interaction domains that may influence HIV management for this population. Further, the qualitative research questions were focused on specific domains, and thus did not examine social interaction processes generally. We took an egocentric approach to assessment of the network, and therefore are limited in inferences to be made about the size and composition of the network as a whole. The study is cross-sectional, which reduces our ability to make causal inferences. Last, because our sample was diverse and about half the cohort was born outside the US/Puerto Rico, the findings may not fully generalize to the broader population of AABL emerging adults living with HIV in the US.

## Conclusions

The present study advances what is known about social networks and other social interaction processes among a diverse cohort of AABL emerging adults living with HIV, including immigrants whose primary language is Spanish and persons with non-suppressed HIV viral load. We found that participants and their social network members exist in constrained socioeconomic contexts, which reduce the resilience of the network in times of challenge. In a previous study, we examined how drug use interferes with HIV viral non-suppression among AABL emerging adults living with HIV [22]. The present study extends this past work to uncover how social networks shape their drug use patterns and how their own drug use shapes their networks. Importantly, we found that a higher proportion of persons in the network using illicit substances is related to unsatisfactory HIV management. Spanish-speaking immigrant AABL emerging adults living with HIV had better HIV outcomes than their peers, raising questions about better understanding resilience among these participants and their networks, and also, in contrast, specific barriers that domestically-born persons living with HIV experience to engagement along the HIV care continuum. The present study highlights intervention targets and strategies to improve the well-being and HIV management abilities of this population, focused on the role of social networks.

## Supplementary Information

Below is the link to the electronic supplementary material.

Supplementary Material 1

Supplementary Material 2

## Data Availability

Data are available upon reasonable request from the corresponding author.

## Abbreviations

AABL: African American/Black and Latine
ACES: Adverse childhood experiences
CDC: Centers for Disease Control and Prevention
IPV: Intimate partner violence
LGBTQ: Lesbian, gay, bisexual, transgender, and queer
MAR: Missing at random
NJ: New Jersey
OR: Odds ratio
US: United States

## References

[1] Centers for Disease Control and Prevention. Ending the HIV epidemic Atlanta, GA: CDC. 2024. Available from: https://www.cdc.gov/ehe/index.html

[2] Frescura L, Godfrey-Faussett P, Feizzadeh AA, El-Sadr W, Syarif O, Ghys PD, et al. Achieving the 95 95 95 targets for all: A pathway to ending AIDS. PLoS ONE. 2022;17(8):e0272405. 35925943 10.1371/journal.pone.0272405 PMC9352102

[3] UNAIDS. Understanding measures of progress towards the 95-95-95 HIV testing, treatment and viral suppression targets. 2023.

[4] Doshi RK, Milberg J, Isenberg D, Matthews T, Malitz F, Matosky M, et al. High rates of retention and viral suppression in the US HIV safety net system: HIV care continuum in the Ryan white HIV/AIDS Program, 2011. Clin Infect Dis. 2015;60(1):117–25. 25225233 10.1093/cid/ciu722

[5] Rangel MC, Gavin L, Reed C, Fowler MG, Lee LM. Epidemiology of HIV and AIDS among adolescents and young adults in the united States. J Adolesc Health. 2006;39(2):156–63. 16857526 10.1016/j.jadohealth.2006.02.011

[6] Centers for Disease Control and Prevention. HIV Surveillance Report. 2017. 2018.

[7] Centers for Disease Control and Prevention. HV surveillance supplemental report: Monitoring selected national HIV prevention and care objectives by using HIV surveillance data United States and 6 territories and freely associated atates, 2022: CDC Stacks. 2024 [updated 21/2024. Available from: https://stacks.cdc.gov/view//156511

[8] Crepaz N, Dong X, Hess KL, Bosh K. Brief report: Racial and ethnic disparities in sustained viral suppression and transmission risk potential among persons aged 13–29 years living with diagnosed HIV infection, united States, 2016. J Acquir Immune Defic Syndr. 2020;83(4):334–9. 31904704 10.1097/QAI.0000000000002277 PMC7055528

[9] Gwadz M, Wilton L, Cleland CM, Serrano S, Sherpa D, Zaldivar MF, et al. A mixed methods descriptive study of a diverse cohort of African American/Black and Latine young and emerging adults living with HIV: Sociodemographic, background, and contextual factors. BMC Public Health. 2025;25(1):620. 39953496 10.1186/s12889-025-21869-3 PMC11829469

[10] Serrano S, Wilton L, Sherpa D, Cleland CM, Zaldivar MF, Maria ZK, et al. Engaging diverse African American/Black and Latine youth and emerging adults living with HIV into research: description of recruitment strategies and lessons learned. AIDS Behav. 2025;29(1):356–76. 39395069 10.1007/s10461-024-04524-7 PMC12977200

[11] Arnett JJ. The developmental context of substance use in emerging adulthood. J Drug Issues. 2005;35(2):235–54.

[12] Osgood DW, Foster EM, Courtney ME. Vulnerable populations and the transition to adulthood. Future Child. 2010;20(1):209–29. 20364628 10.1353/foc.0.0047

[13] Saberi P, Ming K, Dawson-Rose C. What does it mean to be youth-friendly? Results from qualitative interviews with health care providers and clinic staff serving youth and young adults living with HIV. Adolesc Health Med Ther. 2018;9:65–75. 29731672 10.2147/AHMT.S158759 PMC5927154

[14] Logie CH, Wang Y, Lacombe-Duncan A, Wagner AC, Kaida A, Conway T, et al. HIV-related stigma, Racial discrimination, and gender discrimination: pathways to physical and mental health-related quality of life among a National cohort of women living with HIV. Prev Med. 2018;107:36–44. 29277410 10.1016/j.ypmed.2017.12.018

[15] Algarin AB, Zhou Z, Cook CL, Cook RL, Ibanez GE, Age. Sex, Race, Ethnicity, sexual orientation: intersectionality of Marginalized-Group identities and enacted HIV-Related stigma among people living with HIV in Florida. AIDS Behav. 2019;23(11):2992–3001. 31392442 10.1007/s10461-019-02629-y PMC6803104

[16] da Silva DT, Bouris A, Voisin D, Hotton A, Brewer R, Schneider J. Social networks moderate the syndemic effect of psychosocial and structural factors on HIV risk among young black transgender women and men who have sex with men. AIDS Behav. 2020;24(1):192–205. 31289985 10.1007/s10461-019-02575-9 PMC7263264

[17] Harling G, Tsai AC. Using social networks to understand and overcome implementation barriers in the global HIV response. JAIDS: J Acquir Immune Defic Syndr. 2019;82:S244–52. 31764260 10.1097/QAI.0000000000002203 PMC6923140

[18] Tobin KE, Latkin CA. Social networks of HIV positive gay men: their role and importance in HIV prevention. In: Wilton L, editor. Understanding prevention for HIV positive gay men. Springer; 2017. pp. 349–66.

[19] Ewart CK. Social action theory for a public health psychology. Am Psychol. 1991;46(9):931–46. 1958012 10.1037//0003-066x.46.9.931

[20] Holloway IW, Traube DE, Schrager SM, Tan D, Dunlap S, Kipke MD. Psychological distress, health protection, and sexual practices among young men who have sex with men: using social action theory to guide HIV prevention efforts. PLoS ONE. 2017;12(9):e0184482. 28886128 10.1371/journal.pone.0184482 PMC5590937

[21] Traube DE, Holloway IW, Schrager SM, Kipke MD. Utilizing social action theory as a framework to determine correlates of illicit drug use among young men who have sex with men. Psychol Addict Behav. 2012;26(1):78–88. 21644802 10.1037/a0024191 PMC3241957

[22] Wilton L, Gwadz M, Cleland CM, Campos S, Munson MR, Dorsen C, et al. Understanding African American/Black and Latine young and emerging adults living with HIV: a sequential explanatory mixed methods study focused on self-regulatory resources. Int J Equity Health. 2025;24(1). 10.1186/s12939-025-02492-5 PMC12051309 40325383

[23] Skaathun B, Voisin DR, Cornwell B, Lauderdale DS, Schneider JA. A longitudinal examination of factors associated with network bridging among YMSM: implications for HIV prevention. AIDS Behav. 2019;23(5):1326–38. 30136156 10.1007/s10461-018-2258-3 PMC6386635

[24] McFadden RB, Bouris AM, Voisin DR, Glick NR, Schneider JA. Dynamic social support networks of younger black men who have sex with men with new HIV infection. AIDS Care. 2014;26(10):1275–82. 24766079 10.1080/09540121.2014.911807 PMC4087064

[25] Hermanstyne KA, Green HD Jr., Cook R, Tieu HV, Dyer TV, Hucks-Ortiz C, et al. Social network support and decreased risk of seroconversion in black MSM: results of the BROTHERS (HPTN 061) study. J Acquir Immune Defic Syndr. 2018;78(2):163–8. 29424789 10.1097/QAI.0000000000001645 PMC5953785

[26] Valente TW, Gallaher P, Mouttapa M. Using social networks to understand and prevent substance use: A transdisciplinary perspective. Subst Use Misuse. 2004;39(10–12):1685–712. 15587948 10.1081/ja-200033210

[27] Meisel MK, Clifton AD, MacKillop J, Goodie AS. A social network analysis approach to alcohol use and co-occurring addictive behavior in young adults. Addict Behav. 2015;51:72–9. 26240940 10.1016/j.addbeh.2015.07.009

[28] Tucker JA, Cheong J, Chandler SD, Crawford SM, Simpson CA. Social networks and substance use among at-risk emerging adults living in disadvantaged urban areas in the Southern united states: A cross‐sectional naturalistic study. Addiction. 2015;110(9):1524–32. 26054041 10.1111/add.13010

[29] Latkin CA, Van Tieu H, Fields S, Hanscom BS, Connor M, Hanscom B, et al. Social network factors as correlates and predictors of high depressive symptoms among black men who have sex with men in HPTN 061. AIDS Behav. 2017;21(4):1163–70. 27480454 10.1007/s10461-016-1493-8 PMC5288401

[30] Blashill AJ, Perry N, Safren SA. Mental health: A focus on stress, coping, and mental illness as it relates to treatment retention, adherence, and other health outcomes. Curr HIV/AIDS Rep. 2011;8(4):215–22. 21822626 10.1007/s11904-011-0089-1 PMC3623665

[31] Martinez J, Harper G, Carleton RA, Hosek S, Bojan K, Glum G, et al. The impact of stigma on medication adherence among HIV-positive adolescent and young adult females and the moderating effects of coping and satisfaction with health care. AIDS Patient Care STDS. 2012;26(2):108–15. 22149767 10.1089/apc.2011.0178 PMC3266519

[32] Bodenlos JS, Grothe KB, Whitehead D, Konkle-Parker DJ, Jones GN, Brantley PJ. Attitudes toward health care providers and appointment attendance in HIV/AIDS patients. J Assoc Nurses AIDS Care. 2007;18(3):65–73. 17570301 10.1016/j.jana.2007.03.002

[33] Mûnene E, Ekman B. Association between patient engagement in HIV care and antiretroviral therapy medication adherence: cross-sectional evidence from a regional HIV care center in Kenya. AIDS Care. 2015;27(3):378–86. 25298265 10.1080/09540121.2014.963020

[34] Yatchmenoff DK. Measuring client engagement from the client’s perspective in nonvoluntary child protective services. Res Social Work Pract. 2005;15(2):84–96.

[35] Wood TJ, Koester KA, Christopoulos KA, Sauceda JA, Neilands TB, Johnson MO. If someone cares about you, you are more apt to come around: improving HIV care engagement by strengthening the patient–provider relationship. Patient Prefer Adherence. 2018;919 – 27. 10.2147/PPA.S157003 PMC5973398 29872277

[36] Dawson-Rose C, Cuca YP, Webel AR, Solis Baez SS, Holzemer WL, Rivero-Mendez M, et al. Building trust and relationships between patients and providers: an essential complement to health literacy in HIV care. J Assoc Nurses AIDS Care. 2016;27(5):574–84. 27080926 10.1016/j.jana.2016.03.001 PMC5207494

[37] Boulware LE, Cooper LA, Ratner LE, LaVeist TA, Powe NR. Race and trust in the health care system. Public Health Rep. 2003;118(4):358–65. 12815085 10.1016/S0033-3549(04)50262-5 PMC1497554

[38] Levison JH, Bogart LM, Khan IF, Mejia D, Amaro H, Alegría M, et al. Where it falls apart: barriers to retention in HIV care in Latino immigrants and migrants. AIDS Patient Care STDS. 2017;31(9):394–405. 28891715 10.1089/apc.2017.0084 PMC5610398

[39] Saha S, Jacobs EA, Moore RD, Beach MC. Trust in physicians and Racial disparities in HIV care. AIDS Patient Care STDS. 2010;24(7):415–20. 20578909 10.1089/apc.2009.0288 PMC3472674

[40] Westergaard RP, Beach MC, Saha S, Jacobs EA. Racial/ethnic differences in trust in health care: HIV conspiracy beliefs and vaccine research participation. J Gen Intern Med. 2014;29(1):140–6. 23979684 10.1007/s11606-013-2554-6 PMC3889971

[41] Creswell J, Plano Clark V. Designing and conducting mixed methods research. 3rd ed. Thousand Oaks, CA: SAGE Publications Inc.; 2017.

[42] Smith JA, Flowers P, Larkin M. Interpretative phenomenological analysis. 2009.

[43] Fetters MD, Curry LA, Creswell JW. Achieving integration in mixed methods designs-principles and practices. Health Serv Res. 2013;48(6 Pt 2):2134–56. 24279835 10.1111/1475-6773.12117 PMC4097839

[44] National Institutes of Health. All of Us research program 2020. Available from: www.allofus.nih.gov

[45] Hays L, McSweeney J, Mitchell A, Bricker C, Green A, Landes RD. Recruitment issues in emerging adult populations: focus on adult congenital heart disease. Nurs Rep. 2020;10(2):135–45. 34968358 10.3390/nursrep10020017 PMC8608111

[46] DeMonte J, McCumber M, Slye N, Amico KR, Arnold EM, Comulada WS, et al. Adolescents living with or at risk for HIV: A pooled descriptive analysis of studies from the adolescent medicine trials network for HIV/AIDS interventions. J Adolesc Health. 2023;72(5):712–21. 36803999 10.1016/j.jadohealth.2022.12.009 PMC10121857

[47] Lally MA, van den Berg JJ, Westfall AO, Rudy BJ, Hosek SG, Fortenberry JD, et al. HIV continuum of care for youth in the united States. J Acquir Immune Defic Syndromes: JAIDS. 2018;77(1):110–7. 10.1097/QAI.0000000000001563 PMC5774627 28991884

[48] Kahana SY, Jenkins RA, Bruce D, Fernandez MI, Hightow-Weidman LB, Bauermeister JA, et al. Structural determinants of antiretroviral therapy use, HIV care attendance, and viral suppression among adolescents and young adults living with HIV. PLoS ONE. 2016;11(4). 10.1371/journal.pone.0151106 PMC4817971 27035905

[49] Macapagal K, Bhatia R, Greene GJ. Differences in healthcare access, use, and experiences within a community sample of Racially diverse lesbian, gay, bisexual, transgender, and questioning emerging adults. LGBT Health. 2016;3(6):434–42. 27726496 10.1089/lgbt.2015.0124 PMC5165667

[50] Stern MJ, Fordyce E, Hansen C, Heim Viox M, Michaels S, Schlissel A, et al. Social media recruitment for a web survey of sexual and gender minority youth: an evaluation of methods used and resulting sample diversity. LGBT Health. 2020;7(8):448–56. 33147121 10.1089/lgbt.2019.0311

[51] Malterud K, Siersma VD, Guassora AD. Sample size in qualitative interview wtudies: guided by information power. Qual Health Res. 2016;26(13):1753–60. 26613970 10.1177/1049732315617444

[52] McCumber M, Cain D, LeGrand S, Mayer KH, Murphy DA, Psioda MA, et al. Adolescent medicine trials network for HIV/AIDS interventions data harmonization: rationale and development of guidelines. JMIR Res Protocols. 2018;7(12):e11207. 10.2196/11207 PMC6320398 30578242

[53] Gwadz MV, Cleland CM, Leonard NR, Bolas J, Ritchie AS, Tabac L, et al. Understanding organizations for runaway and homeless youth: A multi-setting quantitative study of their characteristics and effects. Child Youth Serv Rev. 2017;73:398–410.

[54] Finkelhor D, Shattuck A, Turner H, Hamby S. A revised inventory of adverse childhood experiences. Child Abuse Negl. 2015;48:13–21. 26259971 10.1016/j.chiabu.2015.07.011

[55] Sherbourne CD, Stewart AL. The MOS social support survey. Soc Sci Med. 1991;32(6):705–14. 2035047 10.1016/0277-9536(91)90150-b

[56] Goldenberg T, Jadwin-Cakmak L, Harper GW. Intimate partner violence among transgender youth: associations with intrapersonal and structural factors. Violence Gend. 2018;5(1):19–25. 29588911 10.1089/vio.2017.0041 PMC5865252

[57] Anderson LA, Dedrick RF. Development of the trust in physician scale: A measure to assess interpersonal trust in patient-physician relationships. Psychol Rep. 1990;67(3f):1091–100. 2084735 10.2466/pr0.1990.67.3f.1091

[58] Mugavero MJ, Westfall AO, Zinski A, Davila J, Drainoni ML, Gardner LI, et al. Measuring retention in HIV care: the elusive gold standard. J Acquir Immune Defic Syndr. 2012;61(5):574–80. 23011397 10.1097/QAI.0b013e318273762f PMC3508092

[59] Yehia BR, Fleishman JA, Metlay JP, Korthuis PT, Agwu AL, Berry SA, et al. Comparing different measures of retention in outpatient HIV care. Aids. 2012;26(9):1131–9. 22382143 10.1097/QAD.0b013e3283528afa PMC3355231

[60] HIV.gov. Seeing your health care provider 2024 [Available from: https://www.hiv.gov/hiv-basics/staying-in-hiv-care/provider-visits-and-lab-test/seeing-your-health-care-provider#:~:text=Current%20HIV%20treatment%20guidelines%20recommend,load%20is%20high%20or%20detectable

[61] Mugavero MJ, Lin HY, Willig JH, Westfall AO, Ulett KB, Routman JS, et al. Missed visits and mortality among patients Establishing initial outpatient HIV treatment. Clin Infect Dis. 2009;48(2):248–56. 19072715 10.1086/595705 PMC2737584

[62] HIV.gov. HIV/AIDS Glossary 2024 [Available from: https://clinicalinfo.hiv.gov/en/glossary/log10#:~:text=A%20mathematical%20term%20used%20to,%2C%20or%202%2C000%2C000%20copies%2FmL

[63] Buuren Sv, Groothuis-Oudshoorn K. Mice: multivariate imputation by chained equations in R. J Stat Softw. 2011;45(3).

[64] Rubin D. Multiple imputation for nonresponse in surveys. Wiley; 1987.

[65] R Core Team. R: A Language and environment for statistical computing. Vienna, Austria: R Foundation for Statistical Computing; 2025.

[66] Hsieh HF, Shannon SE. Three approaches to qualitative content analysis. Qual Health Res. 2005;15(9):1277–88. 16204405 10.1177/1049732305276687

[67] Padgett DK. Qualitative methods in social work research. 3rd ed. Los Angeles, CA: Sage; 2016.

[68] Bourke B, Positionality. Reflecting on the research process. Qualitative Rep. 2014;19(33):1–9.

[69] Milner IVHR. Race, culture, and researcher positionality: working through dangers seen, unseen, and unforeseen. Educational researcher (Washington, DC: 1972). 2007;36(7):388–400.

[70] Smith KP, Christakis NA. Social networks and health. Ann Rev Sociol. 2008;34(34, 2008):405–29.

[71] Arnett JJ. Emerging adulthood: A theory of development from the late teens through the twenties. Am Psychol. 2000;55(5):469. 10842426

[72] Guassi Moreira JF, Tashjian SM, Galván A, Silvers JA. Parents versus peers: assessing the impact of social agents on decision making in young adults. Psychol Sci. 2018;29(9):1526–39. 30088777 10.1177/0956797618778497

[73] García M, Ramos SR, Aponte-Soto L, Ritchwood TD, Drabble LA. Family before anyone else: A qualitative study on family, marginalization, and HIV among Hispanic or Latino/a/x Mexican sexual minority males. Int J Environ Res Public Health. 2022;19(15):8899. 35897270 10.3390/ijerph19158899 PMC9332740

[74] Ryan C, Huebner D, Diaz RM, Sanchez J. Family rejection as a predictor of negative health outcomes in white and Latino lesbian, gay, and bisexual young adults. Pediatrics. 2009;123(1):346–52. 19117902 10.1542/peds.2007-3524

[75] Grieb SM, Kerrigan D, Tepper V, Ellen J, Sibinga E. The clinic environment as a form of social support for adolescents and young adults living with HIV. AIDS Patient Care STDS. 2018;32(5):208–13. 29688746 10.1089/apc.2018.0012 PMC5953214

[76] Tracy EM, Munson MR, Peterson LT, Floersch JE. Social support: A mixed blessing for women in substance abuse treatment. J Social Work Pract Addictions. 2010;10(3):257–82. 10.1080/1533256X.2010.500970 PMC2952953 20953326

[77] Kossinets G, Watts DJ. Empirical analysis of an evolving social network. Science. 2006;311(5757):88–90. 16400149 10.1126/science.1116869

[78] Mowbray O, Quinn A, Cranford JA. Social networks and alcohol use disorders: findings from a nationally representative sample. Am J Drug Alcohol Abuse. 2014;40(3):181–6. 24405256 10.3109/00952990.2013.860984 PMC4004646

[79] Mowbray O, Scott JA. The effect of drug use disorder onset, remission or persistence on an individual’s personal social network. Am J Addictions. 2015;24(5):427–34. 10.1111/ajad.12224 25846575

[80] Wilson N, McDaid S. The mental health effects of a universal basic income: A synthesis of the evidence from previous pilots. Soc Sci Med. 2021;287:114374. 34534779 10.1016/j.socscimed.2021.114374

[81] McKay FH, Bennett R, Dunn M. How, why and for whom does a basic income contribute to health and wellbeing: a systematic review. Health Promot Int. 2023;38(5). 10.1093/heapro/daad119 37804514

[82] Soria C, Lawton L. Connecting fathers: fathers’ impact on adult children’s social networks. Int J Aging Hum Dev. 2023;96(1):19–32. 35698745 10.1177/00914150221106645 PMC9633337

[83] Peterson JL, Rintamaki LS, Brashers DE, Goldsmith DJ, Neidig JL. The forms and functions of peer social support for people living with HIV. J Assoc Nurses AIDS Care. 2012;23(4):294–305. 22079673 10.1016/j.jana.2011.08.014 PMC3303966

[84] Huebner DM, Rullo JE, Thoma BC, McGarrity LA, Mackenzie J. Piloting lead with love: A film-based intervention to improve parents’ responses to their lesbian, gay, and bisexual children. J Prim Prev. 2013;34(5):359–69. 23943135 10.1007/s10935-013-0319-y PMC3797526

[85] Newcomb ME, LaSala MC, Bouris A, Mustanski B, Prado G, Schrager SM, et al. The influence of families on LGBTQ youth health: A call to action for innovation in research and intervention development. LGBT Health. 2019;6(4):139–45. 30844341 10.1089/lgbt.2018.0157 PMC6551980

[86] El-Krab R, Kalichman MO, Eaton LA, Shkembi B, Kalichman SC. Diminished social support as an explanatory mechanism in the relationship between stigma and engagement HIV care. J Behav Med. 2025. 10.1007/s10865-025-00604-8 10.1007/s10865-025-00604-8 41006892

[87] Ma PHX, Chan ZCY, Loke AY. Self-stigma reduction interventions for people living with HIV/AIDS and their families: A systematic review. AIDS Behav. 2019;23(3):707–41. 30298241 10.1007/s10461-018-2304-1

[88] Hunter RF, de la Haye K, Murray JM, Badham J, Valente TW, Clarke M, et al. Social network interventions for health behaviours and outcomes: A systematic review and meta-analysis. PLoS Med. 2019;16(9):e1002890. 31479454 10.1371/journal.pmed.1002890 PMC6719831

[89] Rice SM, Goodall J, Hetrick SE, Parker AG, Gilbertson T, Amminger GP, et al. Online and social networking interventions for the treatment of depression in young people: A systematic review. J Med Internet Res. 2014;16(9):e206. 25226790 10.2196/jmir.3304 PMC4180352

[90] Sansom-Daly UM, Peate M, Wakefield CE, Bryant RA, Cohn RJ. A systematic review of psychological interventions for adolescents and young adults living with chronic illness. Health Psychol. 2012;31(3):380–93. 22059621 10.1037/a0025977

[91] Rhoades H, La Motte-Kerr W, Duan L, Woo D, Rice E, Henwood B, et al. Social networks and substance use after transitioning into permanentsupportive housing. Drug Alcohol Depend. 2018;191:63–9. 30086424 10.1016/j.drugalcdep.2018.06.027 PMC6224132

[92] Ghosh D, Krishnan A, Gibson B, Brown S-E, Latkin CA, Altice FL. Social network strategies to address HIV prevention and treatment continuum of care among at-risk and HIV-infected substance users: A systematic scoping review. AIDS Behav. 2017;21(4):1183–207. 27125244 10.1007/s10461-016-1413-y PMC5085887

[93] Falade-Nwulia O, Felsher M, Kidorf M, Tobin K, Yang C, Latkin C. The impact of social network dynamics on engagement in drug use reduction programs among men and women who use drugs. J Subst Abuse Treat. 2022;137:108713. 34969578 10.1016/j.jsat.2021.108713 PMC9086095

[94] Osilla KC, Kennedy DP, Hunter SB, Maksabedian E. Feasibility of a computer-assisted social network motivational interviewing intervention for substance use and HIV risk behaviors for housing first residents. Addict Sci Clin Pract. 2016;11(1):14. 27604543 10.1186/s13722-016-0061-x PMC5015231

