# Supplementary material for: Social processes and engagement along the HIV care continuum: a mixed methods exploratory study with diverse African American/Black and Latine emerging adults living with HIV
Source: Int J Equity Health. 2025 Oct 28;24:295. doi: 10.1186/s12939-025-02662-5 (PMC12560509; doi:10.1186/s12939-025-02662-5)
Supplement: Supplementary file 2 — Supplementary Material 2 [file 12939_2025_2662_MOESM2_ESM.docx]

**NYU N4 CONNECT**

**LIFE HISTORY Interview Guide**

**LINEAR INTERVIEW**

**Jan 11, 2024**

**INSTRUCTIONS TO INTERVIEWER:**

PART 1 (LIFE EVENTS): The interviewer will create a timeline and note the year during which major events occurred to help guide the discussion. You do not need to include a written timeline with the interview, which should be audio-recorded if the participant agrees. Sketch out the chronological timeline and then go into more detail around important events.

We are interested in the social context and other contextual factors, and how participants managed or coped with important life events.

**Focus on developmental periods**

**0-5 years**

**6-12 years**

**13-16 years**

**17-21 years**

**22 years – present**

PART 2: Then, go back as needed to focus in on HIV medication stops and starts, and the context of stops and starts. Why do people start and stop?

PART 3: Elicit REFLECTIONS on the life course

**Note: As appropriate you can probe for issues around INTERSECTIONAL SYSTEMS OF OPPRESSION and their effects such as racism, classism, sexism, anti-trans sentiment, anti-LGBTQ sentiment, etc**

**INTERVIEW GUIDE**

**Introduction: This is an interview about your life history. Life history interviews are where we talk to people about the major events in their lives and the context of those events. This will include your education, times you moved houses or to different locations, jobs, major relationships, coming out (if you did that), and aspects of HIV.**

**We want to understand you as a whole person and understand your life.**

**You can decline to answer any question you do not wish to ask. Just say “pass.”**

**PART 1 (MAJOR EVENTS WITHIN EACH PERIOD)**

**Focus on developmental periods**

**0-5 years**

**6-12 years**

**13-16 years**

**17-21 years**

**22 years– present**

Think about the period (YEAR TO YEAR). What are some major events that have taken place in this period, events that we’d need to know about to understand you? PROBES CAN INCLUDE

- Family events
- Times moved home or moved to a new country
- Education (graduations)
- First romantic relationship
- Coming out experiences (there may be several)
- Diagnosis with HIV
- Times homeless or unstably housed
- Parenthood
- Other

EXPLORE MAJOR EVENTS, PROBES INCLUDE

Describe the event

Who was involved? (social relationships)

How did it affect you?

How did it affect those around you? (friends, family)

How did you manage it or cope with it?

Anything else we should know?

**PART 2 (HIV INCLUDING STOPS AND STARTS)**

**PART 2: HIV MEDICATION STOPS AND STARTS (add more detail to the timeline)**

Instruction: We are interested in why people take HIV medication for periods of time, and why they stop. We have some questions about that.

1. BRIEFLY First can you talk to me about when you were first diagnosed with HIV? PROBES

Where was that?

Circumstances

Healthcare access and quality

Who told? Who supported?

How started to cope and adapt?

Were you offered HIV medication? What did you think about that?

2. When did you first start HIV medication? PROBES INCLUDE

Circumstances

Side effects

How long stayed on it?

What helped stay on it? What got in the way?

How did you understand or think of adherence at that time? How well-informed did you feel about HIV medication at this time?

Did you want to achieve HIV viral suppression? What do you think about that?

3. ADD TO TIMELINE MAJOR PERIODS OF TAKING HIV MEDICATION AND MAJOR PERIODS NOT TAKING MEDICATION. (GOING FROM PERIOD OF STARTING OR STOPPING TO CONTEXT/EVENTS)

EXPLORE the context of all or most of these periods?

What helped participant stay on it? What got in the way?

4. REFER BACK TO MAJOR EVENTS AND LINK THEM TO STOPPING, STARTING, OR SUSTAINING HIV MEDICATION, AS APPROPRIATE

FOR MAJOR LIFE EVENTS ALREADY REPORTED

Now I want to ask you about major events in your life and how this may or may not have affected you taking your HIV medication.

Think of the major events in your life – big changes in your life related to moving, family, money, work, school, relationships, etc. Do some come to mind?

*Wait for answer.*

*If no:* We want to know more about you and the important changes in your life. What do you think is the biggest thing that happened in your life most recently?

*If yes:* Think of the most recent big event and/or change in your life.

When was it?

What was it?

*Participant describes event.*

Can you tell me more about [insert event here]?

Were you taking HIV medication during this time?

*If yes*: Can you tell me more about this? What helped you keep taking your medication?

- Were you undetectable before this event/change? How about afterwards?

*If no*: why not? *Probe for barriers.*

- How often did you miss doses during this time?
- How regularly were you taking your medication up until this point?
- Were you undetectable before this event/change? How about afterwards?

Were you getting medical care during this time? For example, seeing a doctor or going to a clinic?

*If yes*: Can you tell me more about this? What helped you continue getting medical care?

*If no*: Why not? *Probe for barriers.*

OTHER EVENTS
Are there any other big event/changes that have happened in your life?

Are there any events that interfered with you taking your HIV medication and/or getting medical care?

- If yes, probe for details and barriers to taking HIV medication

**PART 3**

PART 3: We have talked a lot about your whole life. Is there anything you would like to add or emphasize that we may have not covered during this interview? (ELICIT REFLECTIONS, HIGHLIGHT STRENGTHS)

CONCLUDE: You shared a lot of important and personal information today. Do you feel OK to end this interview?

MAKE COUNSELING REFERRALS AS NEEDED
